# Prevalence and associated factors of non-communicable diseases among men: A cross-sectional analysis of Kenya Demographic and Health Survey 2022 data

**DOI:** 10.1371/journal.pone.0327266

**Published:** 2026-07-20

**Authors:** John Baptist Asiimwe, Lilian Nuwabaine, Angella Namulema, Quraish Sserwanja, Joseph Kawuki, Grace Nambozi

**Affiliations:** 1 School of Nursing and Midwifery, Aga Khan University, Kampala, Uganda; 2 Africa Health Sciences University, Kigali, Rwanda; 3 Mbarara Regional Referral Hospital, Mbarara, Uganda; 4 Programmes Department, Relief International, Khartoum, Sudan; 5 Department of Family, Population, and Preventive Medicine, Stony Brook University, New York, United States of America; 6 Department of Nursing, Mbarara University of Science and Technology; University of Environment and Sustainable Development, GHANA

## Abstract

**Background:**

Non-communicable diseases (NCDs) are the leading cause of morbidity and mortality worldwide, with low- and middle-income countries (LMICs) increasingly being disproportionately affected. Although anecdotal evidence or reports indicate an increasing number of Kenyan men having NCDs, the prevalence and associated factors are not well understood. Therefore, this study aimed to determine the prevalence and associated factors of non-communicable diseases among men in Kenya.

**Methods:**

Secondary data comprising 14,439 men aged 15–54 years from the 2022 cross-sectional Demographic and Health Survey (KDHS) in Kenya were analyzed using univariable and multivariable logistic regression analyses in SPSS, version 29.

**Results:**

Overall, the percentage of men with at least one NCD was 9.4% (95% confidence interval [CI]: 8.7–10.2%). Whereas the proportion of participants with multiple NCDs was 1.9 (95% CI: 1.6–2.3). Across NCDs, the highest prevalent NCD was hypertension (3.5% 95%CI:3.1–3.9) followed by depression (2.2% (95%CI:1.9–2.5), anxiety (1.6% (95%CI:1.3–1.9), arthritis (1.4% (95%CI:1.1–1.6), heart disease (1.2% (95%CI:0.9–1.5), diabetes (1% (95%CI:0.8–1.3), lung disease (1% (95%CI:0.7–1.3), and cancer (0.1% (95%CI:0–0.1). In terms of multiple chronic conditions (multimorbidity), the majority of the participants had diabetes and hypertension (0.5% (95%CI:0.3–0.7) followed by hypertension and depression (0.3% (95%CI:0.2–0.4), hypertension and anxiety (0.3% (95%CI:0.2–0.4), and arthritis and depression (0.2% (95%CI:0.1–0.2). Several factors, such as age, region, residence, ethnicity, education level, health status, wealth index, religion, media access, and living a sedentary lifestyle, were found to be significantly associated with the prevalence of NCDs.

**Conclusion:**

The overall prevalence of NCDs among men is relatively low based on available national data. We found that sociodemographic and lifestyle factors were significantly associated with the prevalence of NCDs. Although causal relationships cannot be inferred from this study, the findings suggest that initiatives such as tailored health education, regular medical checkups, and the promotion of physical activity may be relevant components of broader efforts to support men’s health in relation to non‑communicable diseases (NCDs). Region‑specific policies and culturally sensitive approaches may help address variations in risk factors and observed ethnic disparities. Encouraging healthier lifestyle practices among men of all backgrounds and improving access to integrated healthcare in rural areas may also be important considerations. Further research is needed to explore how media exposure, media content, health messaging, and religious contexts may influence men’s exposure to NCDs in Kenya and other sub-Saharan countries.

## Introduction

Non-communicable diseases (NCDs) are a significant global health challenge, contributing to the top ten causes of death across all income levels [1]. The World Health Organization (WHO) reports that cardiovascular diseases are the leading cause of NCD-related deaths, followed by cancers, chronic respiratory diseases, and diabetes [2]. Low-middle-income countries (LMICs) account for 77% of these deaths [3]. In 2021, NCDs were responsible for at least 43 million deaths, equivalent to 75% of non-pandemic-related deaths globally, and 73% of the deaths were in low- and middle-income countries [4].

In Sub-Saharan Africa (SSA), nearly 30% of NCD deaths occur in individuals under 60 years, compared with 13% in high-income countries [5]. The prevalence of NCDs in SSA has risen steadily, with disability-adjusted life-years (DALYs) associated with NCDs increasing by 67% from 1990 to 2017 [6,7]. Kenya, with a population of 49% male and 51% female, has a life expectancy of 64.7 years for men and 69 years for women (KNBS, 2022). Kenya is transitioning from a focus on communicable diseases, such as malaria, HIV/AIDS, and tuberculosis, to a growing burden of NCDs due to behavioral changes and rural-urban migration [8]. Common NCDs in Kenya include stroke, coronary heart disease, dementia, and certain cancers [9].

The rise of NCDs can be attributed to multiple factors, with behavioral changes being a primary driver [10]. Risk factors such as smoking, physical inactivity, obesity, and hypertension are prevalent in both high-income and low- and middle-income countries, including Kenya and SSA [11]. These risk factors are exacerbated by urbanization, with increased exposure to unhealthy diets, sedentary lifestyles, and alcohol consumption contributing significantly to the rise of NCDs [7]. In Kenya, the urban poor population is particularly vulnerable, as they face increased exposure to these common risk factors [5,12]. Furthermore, gender plays a role in the prevalence of NCDs, as men tend to engage in higher-risk behaviors such as smoking and excessive alcohol consumption, which increase their vulnerability to diseases like cardiovascular conditions and diabetes [13].

Kenya has implemented several measures to address the growing NCD burden. The Non-Communicable Diseases and Injury (NCDI) Poverty Commission in Kenya is tasked with assessing disease burden and identifying health service gaps while prioritizing cost-effective interventions [9]. The country’s response also includes the Kenya National Strategy for the Prevention and Control of Non-Communicable Diseases (2015–2020), which aims to reduce NCD-related mortality through prevention, early detection, and healthcare improvements. Despite these efforts, challenges persist, particularly in the allocation of sufficient resources and funding to NCD interventions. Limited prioritization and financial support hinder the full implementation of strategies designed to address NCDs. In 2018, 37% of DALYs lost in Kenya were attributed to NCDs, underscoring their serious public health impact [14]. The inclusion of NCDs in the Kenya Demographic and Health Survey (KDHS) signals recognition of their growing importance, but there remain gaps in comprehensive data collection and monitoring, which hamper effective policymaking. In this study, we focused exclusively on men for three reasons: (1) male‑specific epidemiological analyses remain limited in sub-Saharan African health literature, as men are often regarded as a homogenous and inherently advantaged group and therefore receive less analytical attention; (2) comparable analyses have already been conducted among Kenyan women of reproductive age [14]. Generating parallel evidence for men is essential for balanced and comprehensive NCD policy formulation, and (3) examining men separately allows us to assess whether the prevalence and determinants of NCDs previously documented among women in Kenya are consistent with those observed in the male population. Therefore, this study aimed to determine the prevalence and associated factors of non-communicable diseases among men in Kenya.

## Methods

### Data source, sample design, and collection

This study made use of the 2022 cross-sectional Demographic and Health Survey (KDHS) in Kenya, which used a two-stage stratified sampling approach. The initial stage included the selection of 1,692 enumeration areas (EAs) or clusters from a master sampling frame comprising 129,067 EAs based on the 2019 Kenya population and housing census, employing equal probability and independent selection [15]. The house listing was then created to establish a sampling frame, which was utilized in the next stage to choose 25 households from each cluster. However, if a cluster had fewer than 25 households, all of them were included in the sample. Ultimately, the survey was carried out in 1691 clusters. The Inner-City Fund (ICF) assisted in the pretesting of the study instruments and in training the data collectors, with data collection occurring between February and July of 2022. All men aged 15–54 years who were regular members of the chosen households or who had spent the previous night in those homes were interviewed in either Swahili or English. [15]. In the households selected for the male survey, a total of 16,552 men aged 15–54 were identified as eligible for individual interviews, of whom 14,453 completed the interview, corresponding to a response rate of 87% [15]. However, 14,439 men were included in the analysis if they: (1) were aged 15–54 years; (2) were usual household members or present the night before the survey; and (3) completed the individual men’s full household questionnaire. Men were excluded if they lacked information on the main covariates required for multivariable analysis (incomplete data). The 2022 KDHS dataset was requested, and we secured written permission to use it from the MEASURE DHS website (https://www.dhsprogram.com/data/available-datasets.cfm). While the dataset includes numerous variables, we focused solely on those that were pertinent and applicable to our research.

### Study variables

#### Dependent/outcome.

The main outcome of this study was NCDs. The study analyzed the prevalence and factors associated with eight NCDs that were self-reported by the DHS participants and included heart disease, diabetes, hypertension, arthritis, anxiety, lung disease, depression, and cancer. During the survey, participants were asked to mention whether a doctor or other healthcare workers had informed them of having any of the above-mentioned NCDs. Men who answered yes to any NCD were coded as 1; otherwise, as 0. Two composite variables were constructed as secondary outcomes of the study, and these included having at least 1 NCD (1NCD/yes vs none/no) and having multiple NCDs (≥2NCDs (yes) vs ≤ 1 (No). In this study, multiple NCDs, or NCD multimorbidity was defined as the co-occurrence of two or more chronic long-term conditions, where no condition holds precedence [16].

#### Independent variables.

**Fig 1:**
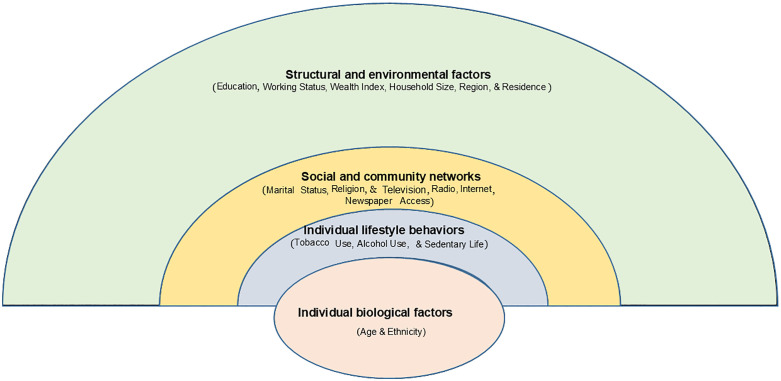
Adapted Social Determinants of Health Model (SDoH) by Dahlgren & Whitehead [17].

The selection and organization of independent variables in this study were guided by Social Determinants of Health Model (SDoH) by Dahlgren & Whitehead [17] (Fig 1). Using the SDoH model we conceptualized the NCD risk as rising from a combination of individual biological factors that are fixed (e.g., age, ethnicity), and other modifiable factors such as the individual lifestyle factors that can promote or damage health (e.g., tobacco use, alcohol use, and physical inactivity), and the social and community networks that may or not provide the NCD related support (e.g., marital status, religion, and access to media channels such as television, radio, internet, and newspaper). Additionally, structural and environmental factors, also termed as living and working conditions, which shape daily life, resource access, and exposure to risks, were included in the analysis, and these included education, working status, wealth index, household size, region, and residence. However, we did not include socioeconomic, cultural, and environmental conditions as these were absent in the dataset.

During analysis, ethnicity was categorized into Kenya’s 12 major tribes: Maasai, Mijikenda/Swahili, Somali, Meru, Taita/Taveta, Embu, Kalenjin, Kamba, Kikuyu, Kisii, Luhya, Luo, and others. Similarly, the region was categorized into Kenya’s eight provinces: the Northeastern, Central, Western, Nairobi, Nyanza, Rift Valley, Eastern, and Coast. Other variables were grouped as follows: religion (Christian, Muslim, or others), marital status (unmarried or married/cohabiting), working status (Yes vs No), household size (≤4 vs ≥ 5 members), education (tertiary, secondary, or none/primary), residence (urban vs rural), age in years (35–54, 25–34, or 15–24), and wealth index (richest, richer, middle, poorer, and poorest). The wealth index was calculated by the 2022 KDHS from information on household asset ownership using principal component analysis [15]. The participants’ perceived health status at the time of the interview (good, moderate, or bad), and exposure to mass media outlets like newspapers, the internet, television, and radio (Yes vs. No) were also considered in the analysis. Three individual lifestyle factors analyzed included: whether the participants consumed alcohol and used tobacco (yes/no), including smoked or smokeless tobacco. The number of hours one spent seated (grouped as high risk (>8 hours)/sedentary life vs. low risk (≤8 hours)/non-sedentary) was also included in the analysis.

### Statistical analysis

The data was analyzed using the complex samples package in SPSS (V29), which addressed the complex sample design inherent in DHS data [18, 19]. The complex sample package delivers accurate estimates of parameters since it considers sample weighting, clustering, and stratification that took place during the selection of study participants [18, 19]. Furthermore, to address the unequal sampling probabilities across various strata, correct for nonresponse, and guarantee that the study results are representative, sample weights from the DHS were utilized for all computed frequencies [18, 19]. Prior to the analysis, the data underwent cleaning, and dummy variables were generated. Descriptive statistics, including frequencies, were calculated for all categorical variables at the univariate level. All variables with P-values less than 0.05 in the univariable complex samples’ logistic regression analysis, based on the Wald F test, were included in the subsequent multivariable logistic regression model to determine factors associated with the prevalence of NCDs. The Wald F test was used as the omnibus criterion to assess the significance of predictors in the final model. All crude and adjusted odds ratios were adjusted for the complex sampling design (weights, strata, and primary sampling units) and are presented with their 95% confidence intervals. Additionally, multi-collinearity among all predictor variables in the model was evaluated using a variance inflation factor (VIF), with a threshold of greater than 10 considered significant [18–20]. All the factors fell below the threshold of 5 (S1 Table). Potential confounders were identified a priori based on existing literature and SDoH model and controlled for in the multivariable models and were retained regardless of statistical significance to minimize omitted variable bias. Lastly, variables with substantial missing data (≥15%) such as the number of minutes exercised per week were excluded in the analysis regardless of their clinical significance. Variables with minimal missing data were handled on a case-by-case basis using list-wise deletion of participants with missing data (n = 14). This brought the final sample size to 14,439 from 14,453 participants who were interviewed. The study findings are reported based on the Strengthening the reporting of observational studies in epidemiology (STROBE) guidelines (S2 Table).

### Ethical consideration

No ethical clearance was necessary to examine the secondary data since it is accessible to the public. Nevertheless, authorization to use the 2022 KDHS datasets was secured from MEASURE DHS (https://www.dhsprogram.com/data/available-datasets.cfm). Ethical permission for the research detailed in the datasets was granted by the ICF Institutional Review Board. The Kenya National Bureau of Statistics carried out the study in collaboration with several other partners. Written informed consent was obtained from human participants, and written informed consent was also obtained from legally authorized representatives of minor participants. High international ethical standards are ensured during MEASURE DHS surveys, and the study protocol was implemented following the relevant guidelines.

## Results

### Structural, individual, behavioral, and community characteristics of the study participants

In total, 14,439 men were included in this analysis (Table 1). Most of the participants were aged 15–34 years (66.7%), lived in the rural setting (61.1%), and identified as being from the Rift Valley, Eastern, Central, and Nairobi provinces (67.3%). The majority, identified as being from the Kikuyu, Luhya, Kalenjin, and Kamba tribes of Kenya (58.4%), were Christians by faith (85.5%), unmarried (51.9%), working (78.2%), and had completed utmost secondary education (79.7%). In total, 65.8% of the participants belonged to the middle, richer, and richest quintiles, and 95% lived in households with more than five members. The majority were exposed to mass media, which included radio (87.4%), television (80.1%), the internet (58.8%), and newspapers (39.4%), and described themselves as being in good health condition (84.1%). In terms of lifestyle behaviors, 26.6% of the participating men consumed alcohol, and 13.3% used tobacco. Additionally, the majority (80.5%) of the participants lived a non-sedentary lifestyle (94.8%).

**Table 1:** Individual, behavioral, community, and structural characteristics of the study participants and their NCD status.

	Overall		NCD status		
Variable (N = 14,439)	*n*	Weighted *%*	No NCD	At least 1 NCD	P-value
**Age (years)**					<0.001
15-24	5,577	38.6	5,269	307	
25-34	4,049	28.1	3,763	287	
35-59	4,813	33.3	4,048	765	
**Residence**					0.574
Urban	5,610	38.9	5,066	544	
Rural	8,829	61.1	8,014	815	
**Region/Province**					0.001
Coast	1,341	9.3	1,242	98	
Northeastern	273	1.9	264	9	
Eastern	2,123	14.7	1,937	186	
Central	1,945	13.5	1,756	189	
Rift Valley	3,808	26.4	3,453	354	
Western	1,499	10.4	1,342	156	
Nyanza	1,610	11.2	1,485	125	
Nairobi	1,841	12.7	1,600	240	
**Ethnicity**					<0.001
Embu	186	1.3	168	18	
Kalenjin	1,823	12.6	1,653	170	
Kamba	1,660	11.5	1,582	78	
Kikuyu	2,521	17.5	2,219	303	
Kisii	901	6.2	847	54	
Luhya	2,424	16.8	2,205	219	
Luo	1,591	11.0	1,414	177	
Maasai	322	2.2	298	24	
Meru	717	5.0	591	126	
Mijikenda/Swahili	798	5.5	736	62	
Somali	294	2.0	280	14	
Taita/Taveta	136	0.9	116	20	
Other	1,065	7.4	972	93	
**Education**					<0.001
None/primary	5,657	39.2	5,120	536	
Secondary	5,842	40.5	5,389	453	
Tertiary	2,940	20.4	2,571	369	
**Religion**					0.030
Christians	12,347	85.5	11,208	1,138	
Muslims	1,042	7.2	958	85	
Others	1,050	7.3	914	136	
**Marital status**					<0.001
Unmarried (single, divorced/widowed/separated)	7,490	51.9	6,868	522	
Married/Cohabiting	6,949	48.1	6,112	838	
**Working status/occupation**					<0.001
Not Working	3,157	21.8	2,954	202	
Working	11,282	78.2	10,126	1,157	
**Wealth index**					0.001
Poorest	2,175	15.1	1,999	176	
Poorer	2,762	19.1	2,518	244	
Middle	2,929	20.3	2,689	240	
Richer	3,493	24.2	3,170	323	
Richest	3,079	21.3	2,704	375	
**Household number**					0.230
≤4	714	4.9	658	56	
≥5	13,725	95.1	12,421	1,304	
**Health status**					<0.001
Bad	186	1.3	133	54	
Moderate	2,110	14.6	1,707	403	
Good	12,143	84.1	11,240	903	
**Newspapers**					0.188
No	8,747	60.6	7,957	790	
Yes	5,692	39.4	5,123	569	
**TV**					0.534
No	2,873	19.9	2,614	259	
Yes	11,566	80.1	10,466	1,101	
**Radio**					0.590
No	1,817	12.6	1,635	182	
Yes	12,622	87.4	11,445	1,178	
**Internet use**					0.695
No	5,955	41.2	5,404	551	
Yes	8,484	58.8	7,676	809	
**Lifestyle Behavioral factors**					
**Tobacco use**					<0.001
No	12,517	86.7	11,407	1,110	
Yes	1,922	13.3	1,673	249	
**Number of hours per day seated/sedentary**					0.530
Low risk (≤8 hours)/non-sedentary	13,692	94.8	12,412	1,279	
High risk (>8 hours)/sedentary life	747	5.2	668	79	
**Alcohol consumption**					0.001
No	10,600	73.4	9,688	912	
Yes	3,839	26.6	3,392	447	

### Prevalence of NCDs among men in Kenya

Overall, the percentage of men with at least one NCD was 9.4% (95% CI: 8.7–10.2%, Table 2). Whereas the proportion of participants with multiple NCDs was 1.9% (95% CI: 1.6–2.3). Across NCDs, the highest prevalent NCD was hypertension (3.5% 95%CI:3.1–3.9), followed by depression (2.2% (95%CI:1.9–2.5), anxiety (1.6% (95%CI:1.3–1.9), arthritis (1.4% (95%CI:1.1–1.6), heart disease (1.2% (95%CI:0.9–1.5), diabetes (1% (95%CI:0.8–1.3), lung disease (1% (95%CI:0.7–1.3), and cancer (0.1% (95%CI:0.00–0.1). In terms of multimorbidity, the majority of the participants had diabetes and hypertension (0.5 (95%CI:0.3–0.7) followed by hypertension and depression (0.3 (95%CI:0.2–0.4), hypertension and anxiety (0.3 (95%CI:0.2–0.4) and arthritis and depression (0.2 (95%CI:0.1–0.2).

**Table 2:** Prevalence of NCDs among men in Kenya.

Variable (N = 14,439)	Yes		No	
	*n*	Weighed *%* (95%CI)	*n*	Weighed *%* (95%CI)
Hypertension	504	3.5 (3.1-3.9)	13,935	96.5 (96.1-96.9)
Diabetes	144	1 (0.8-1.3)	14,295	99.0 (98.7-99.2)
Heart disease	172	1.2 (0.9-1.5)	14,267	98.8 (98.5-99.1)
Lung disease	139	1 (0.7-1.3)	14,300	99.0 (98.7-99.3)
Depression	319	2.2 (1.9-2.5)	14,120	97.8 (97.5-98.1)
Anxiety	224	1.5 (1.3-1.9)	14,215	98.5 (98.1-98.7)
Arthritis	197	1.4 (1.1-1.6)	14,242	98.6 (98.4-98.9)
Cancer	8	0.1 (0.00-0.1)	14,431	99.9 (99.9-100.0)
Living with at least 1 NCD	1359	9.4 (8.7-10.2)	13080	90.6 (89.8-91.3)
Living with ≥2 NCDs	279	1.9 (1.6-2.3)	14,160	98.1 (97.7-98.4)
**Multi-morbidity**				
Hypertension & Diabetes	68	0.5 (0.3-0.7)	14,371	96.6 (98.6-100)
Hypertension & Depression	47	0.3 (0.2-0.4)	14,392	99.7 (98.7-100)
Diabetes & Depression	11	0.1 (0.041-0.1)	14,428	99.9 (99.1-100)
Hypertension & Anxiety	39	0.3 (0.2-0.4)	14,400	99.7 (98.7-100)
Diabetes & Anxiety	12	0.1 (0.04-0.2)	14,427	99.9 (99.1-100)
Arthritis & Depression	23	0.2 (0.1-0.2)	14,416	99.9 (99.1-100)
Arthritis & Anxiety	8	0.1 (0.03-0.1)	14,431	99.9 (99.1-100)
Heart Disease & Depression	12	0.1 (0.05-0.2)	14,427	99.9 (99.1-100)
Heart Disease & Anxiety	6	0.04 (0.02-0.1)	14434	99.9 (99.1-100)
Diabetes & Hypertension & Depression	8	0.1 (0.02-0.1)	14,431	99.9 (98.6-100)
Diabetes & Hypertension & Anxiety	9	0.1 (0.02-0.2)	14,430	99.9 (98.8-100)
Diabetes & Hypertension & Depression & Anxiety	3	0.02 (0.01-0.1)	14,436	99.9 (98.7-100)

Note. NCD = non-communicable diseases. Due to a small sample size (n = 8) of those who self-reported having cancer, further analysis of data related to cancer was not undertaken.

### Factors associated with having specific/multiple NCDs

Tables 3 and 4 summarize the results of univariable and multivariable analyses of the factors associated with each specific NCD and having multiple NCDs, respectively. Several factors were found to be associated with having specific and multiple NCDs, which included age, region, residence, ethnicity, education level, health status, wealth index, religion, media access, and living a sedentary lifestyle. We found that compared with younger men (15–24 years), older participants (≥25 years) had a higher likelihood of experiencing hypertension (aOR 2.69 (95%CI: 1.58–4.56), diabetes mellitus (aOR 3.02 (95%CI: 1.19–7.64), arthritis (aOR 4.56 (95%CI:2.87–7.25), depression (aOR 4.31 (95%CI: 2.76–6.73), having atleast one NCD (aOR 2.90 (95%CI:2.13–3.95) and multiple NCDs (aOR 7.17 (95%CI 2.75–18.69).

**Table 3:** Factors associated with having specific/individual NCDs among men in Kenya.

	Hypertension		Diabetes		Heart diseases		Lung Diseases	
**Variable (N = 14,439)**	*uORs (*95 CI)	*aORs (*95 CI)	*uORs (*95 CI)	*aORs (*95 CI)	*uORs (*95 CI)	*aORs (*95 CI)	*uORs (*95 CI)	*aORs (*95 CI)
Age (years)								
15-24	1	1	1	1	1	–	1	–
25-34	2.69 (1.58-4.56)*	1.83 (0.98–3.41)	3.02 (1.19-7.64)**	1.29 (0.47–3.55)	0.65 (0.34-1.22)	–	0.62 (0.24-1.57)	–
35-59	9.63 (6.31-14.71)*	6.94 (3.99–12.08)*	16.13 (7.91-32.90)**	6.84 (3.02–15.50)**	0.71 (0.40-1.25)	–	1.09 (0.51-2.35)	–
**Residence**								–
Urban	1.15 (0.87-1.51)	–	1.31 (0.78-2.20)	–	1.21 (0.69-2.10)	–	1.63 (0.85-3.12)	–
Rural	1	–	1	–	1	–	1	–
**Region/Province**								
Coast	1	–	1	1	1	1	1	1
Northeastern	0.52 (0.26-1.03)	–	1.92 (0.83-4.47)	3.30 (0.87–12.51)	0.53 (0.15-1.83)	6.53 (1.11–38.46)*	0.00 (0.00-0.00)	0.00 (0.00–0.00)
Eastern	0.77 (0.52-1.15)	–	0.94 (0.41-2.17)	0.44 (0.12–1.58)	1.58 (0.64-3.89)	16.12 (1.93–134.76)*	0.58 (0.20-1.73)	0.63 (0.20–2.01)
Central	1.02 (0.67-1.57)	–	2.45 (1.07-5.61)*	0.83 (0.24–2.85)	0.92 (0.33-2.56)	8.44 (0.64–110.67)	1.72 (0.59-4.99)	1.17 (0.38–3.59)
Rift Valley	0.70 (0.49-1.02)	–	0.94 (0.45-1.99)	0.39 (0.15–1.03)	1.26 (0.55-2.85)	6.14 (0.69–54.26)	1.10 (0.44-2.74)	1.06 (0.38–2.95)
Western	0.90 (0.57-1.43)	–	1.34 (0.57-3.17)	1.15 (0.44–3.04)	2.21 (0.94-5.21)	17.72 (1.74–180.26)*	2.98 (1.15-7.70)*	3.22 (1.16–8.96)*
Nyanza	0.92 (0.62-1.37)	–	2.04 (0.97-4.32)	1.06 (0.31–3.59)	1.64 (0.69-3.88)	13.97 (1.12–174.67)*	1.19 (0.44-3.22)	0.65 (0.21–2.00)
Nairobi	1.25 (0.71-2.21)	–	1.97 (0.60-6.40)	0.50 (0.16–1.55)	4.21 (1.61-10.05)**	31.49 (3.26–303.97)*	3.80 (1.07-13.51)*	2.54 (0.75–8.56)
**Ethnicity**								
Embu	1.19 (0.60-2.35)	1.00 (0.44–2.26)	0.46 (0.10-2.05)	0.53 (0.10–2.80)	1.02 (0.20-5.21)	0.54 (0.06–4.50)	0.66 (0.16-2.70)	0.92 (0.22–3.83)
Kalenjin	0.44 (0.28-0.71)**	0.59 (0.36–0.95)**	0.46 (0.23-0.95)*	1.27 (0.45–3.60)	1.64 (0.64-4.21)	2.73 (0.64–11.65)	0.31 (0.13-0.77)*	0.36 (0.12–1.04)
Kamba	0.32 (0.16-0.61)**	0.38 (0.19–0.74)**	0.23 (0.09-0.59)*	0.42 (0.14–1.31)	1.36 (0.48-3.81)	0.87 (0.21–3.63)	0.42 (0.15-1.23)	0.49 (0.15–1.61)
Kikuyu	1	1	1	1	1	1	1	1
Kisii	0.53 (0.31-0.91)**	0.60 (0.35–1.03)	0.59 (0.24-1.47)	0.76 (0.22–2.63)	0.09 (0.01-0.60)*	0.07 (0.01–0.59)*	0.91 (0.15-5.49)	1.05 (0.18–6.07)
Luhya	0.69 (0.45-1.07)	0.82 (0.52–1.31)	0.44 (0.21-0.93)*	0.48 (0.16–1.44)	1.49 (0.61-3.66)	0.84 (0.20–3.56)	0.98 (0.50-1.93)	0.52 (0.24–1.13)
Luo	1.15 (0.69-1.90)	1.06 (0.64–1.76)	0.99 (0.42-2.31)	0.75 (0.15–3.65)	1.90 (0.85-4.26)	0.91 (0.22–3.66)	1.30 (0.50-3.35)	1.09 (0.36–3.28)
Maasai	0.42 (0.14-1.26)	0.74 (0.25–2.20	0.46 (0.10-2.07)	1.89 (0.42–8.46)	1.22 (0.31-4.69)	1.94 (0.36–10.51)	0.34 (0.07-1.57)	0.37 (0.07–2.01)
Meru	1.52 (0.85-2.72)	1.60 (0.93–2.74)	1.41 (0.39-5.15)	1.91 (0.48–7.56)	1.23 (0.39-3.92)	0.71 (0.14–3.58)	0.39 (0.12-1.29)	0.55 (0.16–1.91)
Mijikenda/Swahili	0.91 (0.57-1.47)	1.41 (0.84–2.34)	0.24 (0.07-0.85)*	0.34 (0.06–1.83)	1.14 (0.37-3.56)	11.10 (0.91–134.67)	0.50 (0.13-1.98)	0.95 (0.18–4.96)
Somali	0.64 (0.31-1.31)	1.10 (0.46–2.61)	0.81 (0.39-1.68)	0.72 (0.16–3.19)	0.41 (0.12-1.46)	0.59 (0.10–3.44)	0.16 (0.02-1.20)	1.42 (0.14–14.44)
Taita/Taveta	1.05 (0.50-2.16)	1.07 (0.49–2.36)	0.50 (0.11-2.18)	0.39 (0.08–2.02)	10.79 (1.38-84.17)*	23.63 (1.18–471.56)*	0.34 (0.04-2.70)	0.38 (0.05–3.18)
Other	0.79 (0.46-1.34)	1.05 (0.60–1.84)	0.61 (0.22-1.64)	1.03 (0.25–4.31)	2.36 (0.65-8.61)	1.93 (0.38–9.83)	1.28 (0.38-4.27)	1.15 (0.27–4.99)
**Education**								
None/primary	1	1	1	1	1	–	1	1
Secondary	0.82 (0.60-1.11)	1.18 (0.84–1.65)	0.94 (0.57-1.55)	1.23 (0.72–2.10)	1.22 (0.73-2.03)	–	1.59 (0.93-2.70)*	1.83 (1.09–3.08)*
Tertiary	1.66 (1.20-2.29)**	1.67 (1.11–2.51)*	2.74 (1.55-4.82)**	2.11 (1.15–3.87)*	0.57 (0.25-1.26)	–	2.54 (1.27-5.08)*	2.67 (1.43–5.00)*
**Religion**								
Christians	1	–	1	–	1	–	1	
Muslims	1.17 (0.80-1.70)	–	0.65 (0.35-1.21)	–	0.47 (0.21-1.02)	–	0.31 (0.12-0.76)	
Others	0.58 (0.31-1.08)	–	1.26 (0.53-2.96)	–	0.64 (0.21-1.95)	–	1.77 (0.59-5.32)	
**Marital status**								
Unmarried (single/divorced/widowed/separated)	1	1	1	1	1	–	1	–
Married/Cohabiting	3.70 (2.74-4.99)*	1.27 (0.88–1.82)	**5.84 (3.10-10.99**)**	1.62 (0.85–3.12)	0.90 (0.53-1.54)	–	0.63 (0.34-1.15)	–
**Working status/occupation**								
Not Working	1	1	1	1	1	–	1	–
Working	3.12 (2.03-4.81)**	1.03 (0.64–1.65)	6.13 (2.33-16.15)**	1.93 (0.60–6.24)	0.79 (0.42-1.48)	–	1.41 (0.57-3.45)	–
**Wealth index**								
Poorest	1	1	1	1	1	–	1	–
Poorer	1.02 (0.69-1.52)	0.99 (0.64–1.51)	1.61 (0.63-4.10)	1.42 (0.56–3.65)	1.05 (0.57-1.92)	–	0.93 (0.50-1.76)	–
Middle	1.25 (0.86-1.83)	1.21 (0.79–1.84)	3.13 (1.32-7.42)**	3.09 (1.24–7.67)*	0.80 (0.43-1.47)	–	0.73 (0.36-1.48)	–
Richer	1.40 (0.94-2.08)	1.25 (0.78–2.02)	3.09 (1.31-7.28)**	3.32 (1.31–8.40) *	0.97 (0.46-2.03)	–	1.91 (0.83-4.39)	–
Richest	2.34 (1.55-3.53)**	1.79 (1.04–3.09)*	7.26 (2.98-17.67)**	6.95 (2.76–17.55)*	0.93 (0.40-2.16)	–	0.97 (0.37-2.56)	–
**Household number**								
≤4	1	–	1	–	1	–	1	–
≥5	1.23 (0.68-2.22)	–	0.49 (0.22-1.10)	–	1.83 (0.61-5.52)	–	1.99 (0.78-5.02)	–
**Health status**								
Bad	1	1	1	1	1	1	1	1
Moderate	0.62 (0.35-1.07)	0.59 (0.33–1.05)	0.67 (0.27-1.66)	0.56 (0.20–1.55)	0.91 (0.32-2.60)	0.74 (0.25–2.14)	0.83 (0.30-2.32)	0.77 (0.26–2.33)
Good	0.21 (0.13-0.35)**	0.22 (0.12–0.38)**	0.14 (0.06-0.32)**	0.11 (0.04–0.29)**	0.22 (0.08-0.62)**	0.19 (0.07–0.54)**	0.30 (0.11-0.80)**	0.29 (0.10–0.87)*
**Media access (composite)**								
No	–	–	–	–	–	–	1	1
Yes	–	–	–	–	–	–	0.33 (0.15-0.73)*	0.19 (0.08-0.47)*
**Newspaper**								
No	1	1	1	–	1	–	1	–
Yes	1.36 (1.04-1.79)*	0.97 (0.72–1.31)	1.46 (0.88-2.41)	–	0.68 (0.41-1.14)	–	1.28 (0.80-2.06)	–
**TV**				–				
No	1	1	1	–	1	–	1	–
Yes	1.43 (1.08-1.89)*	1.13 (0.82–1.57)	1.33 (0.78-2.25)	–	0.87 (0.50-1.50)	–	0.60 (0.35-1.03)	–
**Radio**				–		–		
No	1	–	1	–	1	–	1	–
Yes	1.26 (0.83-1.91)	–	0.55 (0.29-1.07)	–	0.94 (0.45-1.99)	–	0.91 (0.44-1.90)	–
**Internet use**						–		
No	1	1	1	1	1	–	1	–
Yes	1.36 (1.03-1.79)*	1.20 (0.82–1.75)	1.63 (1.04-2.57)*	0.97 (0.61–1.52)	0.95 (0.59-1.52)	–	1.01 (0.55-1.85)	–
**Lifestyle Behavioral factors**								
**Tobacco use**								–
No	1	–	1	–	1	–	1	–
Yes	1.30 (0.94-1.79)	–	0.70 (0.32-1.50)	–	0.90 (0.45-1.81)	–	1.36 (0.70-2.64)	–
**Number of hours per day seated/sedentary**								
Low risk (≤8 hours)/non-sedentary life	1	–	1	1	1	–	1	–
High-risk (>8 hours)/sedentary life	1.32 (0.67-2.60)	–	3.41 (1.28-9.09)*	2.90 (1.30-6.46)*	0.75 (0.27-2.08)	–	0.44 (0.17-1.15)	–
**Alcohol consumption**								
No	1	1	1	–	1	–	1	–
Yes	1.63 (1.23-2.17)**	1.16 (0.85–1.57)	1.18 (0.68-2.07)	–	0.90 (0.52-1.55)	–	1.32 (0.67-2.61)	–
**Age (years)**								
15-24	1	1	1	1	1	–	1	–
25-34	2.69 (1.58-4.56)*	1.83 (0.98–3.41)	3.02 (1.19-7.64)**	1.29 (0.47–3.55)	0.65 (0.34-1.22)	–	0.62 (0.24-1.57)	–
35-59	9.63 (6.31-14.71)*	6.94 (3.99–12.08)*	16.13 (7.91-32.90)**	6.84 (3.02–15.50)**	0.71 (0.40-1.25)	–	1.09 (0.51-2.35)	–
**Residence**								–
Urban	1.15 (0.87-1.51)	–	1.31 (0.78-2.20)	–	1.21 (0.69-2.10)	–	1.63 (0.85-3.12)	–
Rural	1	–	1	–	1	–	1	–
**Region/Province**								
Coast	1	–	1	1	1	1	1	1
Northeastern	0.52 (0.26-1.03)	–	1.92 (0.83-4.47)	3.30 (0.87–12.51)	0.53 (0.15-1.83)	6.53 (1.11–38.46)*	0.00 (0.00-0.00)	0.00 (0.00–0.00)
Eastern	0.77 (0.52-1.15)	–	0.94 (0.41-2.17)	0.44 (0.12–1.58)	1.58 (0.64-3.89)	16.12 (1.93–134.76)*	0.58 (0.20-1.73)	0.63 (0.20–2.01)
Central	1.02 (0.67-1.57)	–	2.45 (1.07-5.61)*	0.83 (0.24–2.85)	0.92 (0.33-2.56)	8.44 (0.64–110.67)	1.72 (0.59-4.99)	1.17 (0.38–3.59)
Rift Valley	0.70 (0.49-1.02)	–	0.94 (0.45-1.99)	0.39 (0.15–1.03)	1.26 (0.55-2.85)	6.14 (0.69–54.26)	1.10 (0.44-2.74)	1.06 (0.38–2.95)
Western	0.90 (0.57-1.43)	–	1.34 (0.57-3.17)	1.15 (0.44–3.04)	2.21 (0.94-5.21)	17.72 (1.74–180.26)*	2.98 (1.15-7.70)*	3.22 (1.16–8.96)*
Nyanza	0.92 (0.62-1.37)	–	2.04 (0.97-4.32)	1.06 (0.31–3.59)	1.64 (0.69-3.88)	13.97 (1.12–174.67)*	1.19 (0.44-3.22)	0.65 (0.21–2.00)
Nairobi	1.25 (0.71-2.21)	–	1.97 (0.60-6.40)	0.50 (0.16–1.55)	4.21 (1.61-10.05)**	31.49 (3.26–303.97)*	3.80 (1.07-13.51)*	2.54 (0.75–8.56)
**Ethnicity**								
Embu	1.19 (0.60-2.35)	1.00 (0.44–2.26)	0.46 (0.10-2.05)	0.53 (0.10–2.80)	1.02 (0.20-5.21)	0.54 (0.06–4.50)	0.66 (0.16-2.70)	0.92 (0.22–3.83)
Kalenjin	0.44 (0.28-0.71)**	0.59 (0.36–0.95)**	0.46 (0.23-0.95)*	1.27 (0.45–3.60)	1.64 (0.64-4.21)	2.73 (0.64–11.65)	0.31 (0.13-0.77)*	0.36 (0.12–1.04)
Kamba	0.32 (0.16-0.61)**	0.38 (0.19–0.74)**	0.23 (0.09-0.59)*	0.42 (0.14–1.31)	1.36 (0.48-3.81)	0.87 (0.21–3.63)	0.42 (0.15-1.23)	0.49 (0.15–1.61)
Kikuyu	1	1	1	1	1	1	1	1
Kisii	0.53 (0.31-0.91)**	0.60 (0.35–1.03)	0.59 (0.24-1.47)	0.76 (0.22–2.63)	0.09 (0.01-0.60)*	0.07 (0.01–0.59)*	0.91 (0.15-5.49)	1.05 (0.18–6.07)
Luhya	0.69 (0.45-1.07)	0.82 (0.52–1.31)	0.44 (0.21-0.93)*	0.48 (0.16–1.44)	1.49 (0.61-3.66)	0.84 (0.20–3.56)	0.98 (0.50-1.93)	0.52 (0.24–1.13)
Luo	1.15 (0.69-1.90)	1.06 (0.64–1.76)	0.99 (0.42-2.31)	0.75 (0.15–3.65)	1.90 (0.85-4.26)	0.91 (0.22–3.66)	1.30 (0.50-3.35)	1.09 (0.36–3.28)
Maasai	0.42 (0.14-1.26)	0.74 (0.25–2.20	0.46 (0.10-2.07)	1.89 (0.42–8.46)	1.22 (0.31-4.69)	1.94 (0.36–10.51)	0.34 (0.07-1.57)	0.37 (0.07–2.01)
Meru	1.52 (0.85-2.72)	1.60 (0.93–2.74)	1.41 (0.39-5.15)	1.91 (0.48–7.56)	1.23 (0.39-3.92)	0.71 (0.14–3.58)	0.39 (0.12-1.29)	0.55 (0.16–1.91)
Mijikenda/Swahili	0.91 (0.57-1.47)	1.41 (0.84–2.34)	0.24 (0.07-0.85)*	0.34 (0.06–1.83)	1.14 (0.37-3.56)	11.10 (0.91–134.67)	0.50 (0.13-1.98)	0.95 (0.18–4.96)
Somali	0.64 (0.31-1.31)	1.10 (0.46–2.61)	0.81 (0.39-1.68)	0.72 (0.16–3.19)	0.41 (0.12-1.46)	0.59 (0.10–3.44)	0.16 (0.02-1.20)	1.42 (0.14–14.44)
Taita/Taveta	1.05 (0.50-2.16)	1.07 (0.49–2.36)	0.50 (0.11-2.18)	0.39 (0.08–2.02)	10.79 (1.38-84.17)*	23.63 (1.18–471.56)*	0.34 (0.04-2.70)	0.38 (0.05–3.18)
Other	0.79 (0.46-1.34)	1.05 (0.60–1.84)	0.61 (0.22-1.64)	1.03 (0.25–4.31)	2.36 (0.65-8.61)	1.93 (0.38–9.83)	1.28 (0.38-4.27)	1.15 (0.27–4.99)
**Education**								
None/primary	1	1	1	1	1	–	1	1
Secondary	0.82 (0.60-1.11)	1.18 (0.84–1.65)	0.94 (0.57-1.55)	1.23 (0.72–2.10)	1.22 (0.73-2.03)	–	1.59 (0.93-2.70)*	1.83 (1.09–3.08)*
Tertiary	1.66 (1.20-2.29)**	1.67 (1.11–2.51)*	2.74 (1.55-4.82)**	2.11 (1.15–3.87)*	0.57 (0.25-1.26)	–	2.54 (1.27-5.08)*	2.67 (1.43–5.00)*
**Religion**								
Christians	1	–	1	–	1	–	1	
Muslims	1.17 (0.80-1.70)	–	0.65 (0.35-1.21)	–	0.47 (0.21-1.02)	–	0.31 (0.12-0.76)	
Others	0.58 (0.31-1.08)	–	1.26 (0.53-2.96)	–	0.64 (0.21-1.95)	–	1.77 (0.59-5.32)	
**Marital status**								
Unmarried (single/divorced/widowed/separated)	1	1	1	1	1	–	1	–
Married/Cohabiting	3.70 (2.74-4.99)*	1.27 (0.88–1.82)	**5.84 (3.10-10.99**)**	1.62 (0.85–3.12)	0.90 (0.53-1.54)	–	0.63 (0.34-1.15)	–
**Working status/occupation**								
Not Working	1	1	1	1	1	–	1	–
Working	3.12 (2.03-4.81)**	1.03 (0.64–1.65)	6.13 (2.33-16.15)**	1.93 (0.60–6.24)	0.79 (0.42-1.48)	–	1.41 (0.57-3.45)	–
**Wealth index**								
Poorest	1	1	1	1	1	–	1	–
Poorer	1.02 (0.69-1.52)	0.99 (0.64–1.51)	1.61 (0.63-4.10)	1.42 (0.56–3.65)	1.05 (0.57-1.92)	–	0.93 (0.50-1.76)	–
Middle	1.25 (0.86-1.83)	1.21 (0.79–1.84)	3.13 (1.32-7.42)**	3.09 (1.24–7.67)*	0.80 (0.43-1.47)	–	0.73 (0.36-1.48)	–
Richer	1.40 (0.94-2.08)	1.25 (0.78–2.02)	3.09 (1.31-7.28)**	3.32 (1.31–8.40) *	0.97 (0.46-2.03)	–	1.91 (0.83-4.39)	–
Richest	2.34 (1.55-3.53)**	1.79 (1.04–3.09)*	7.26 (2.98-17.67)**	6.95 (2.76–17.55)*	0.93 (0.40-2.16)	–	0.97 (0.37-2.56)	–
**Household number**								
≤4	1	–	1	–	1	–	1	–
≥5	1.23 (0.68-2.22)	–	0.49 (0.22-1.10)	–	1.83 (0.61-5.52)	–	1.99 (0.78-5.02)	–
**Health status**								
Bad	1	1	1	1	1	1	1	1
Moderate	0.62 (0.35-1.07)	0.59 (0.33–1.05)	0.67 (0.27-1.66)	0.56 (0.20–1.55)	0.91 (0.32-2.60)	0.74 (0.25–2.14)	0.83 (0.30-2.32)	0.77 (0.26–2.33)
Good	0.21 (0.13-0.35)**	0.22 (0.12–0.38)**	0.14 (0.06-0.32)**	0.11 (0.04–0.29)**	0.22 (0.08-0.62)**	0.19 (0.07–0.54)**	0.30 (0.11-0.80)**	0.29 (0.10–0.87)*
**Media access (composite)**								
No	–	–	–	–	–	–	1	1
Yes	–	–	–	–	–	–	0.33 (0.15-0.73)*	0.19 (0.08-0.47)*
**Newspaper**								
No	1	1	1	–	1	–	1	–
Yes	1.36 (1.04-1.79)*	0.97 (0.72–1.31)	1.46 (0.88-2.41)	–	0.68 (0.41-1.14)	–	1.28 (0.80-2.06)	–
**TV**				–				
No	1	1	1	–	1	–	1	–
Yes	1.43 (1.08-1.89)*	1.13 (0.82–1.57)	1.33 (0.78-2.25)	–	0.87 (0.50-1.50)	–	0.60 (0.35-1.03)	–
**Radio**				–		–		
No	1	–	1	–	1	–	1	–
Yes	1.26 (0.83-1.91)	–	0.55 (0.29-1.07)	–	0.94 (0.45-1.99)	–	0.91 (0.44-1.90)	–
**Internet use**						–		
No	1	1	1	1	1	–	1	–
Yes	1.36 (1.03-1.79)*	1.20 (0.82–1.75)	1.63 (1.04-2.57)*	0.97 (0.61–1.52)	0.95 (0.59-1.52)	–	1.01 (0.55-1.85)	–
**Lifestyle Behavioral factors**								
**Tobacco use**								–
No	1	–	1	–	1	–	1	–
Yes	1.30 (0.94-1.79)	–	0.70 (0.32-1.50)	–	0.90 (0.45-1.81)	–	1.36 (0.70-2.64)	–
**Number of hours per day seated/sedentary**								
Low risk (≤8 hours)/non-sedentary life	1	–	1	1	1	–	1	–
High-risk (>8 hours)/sedentary life	1.32 (0.67-2.60)	–	3.41 (1.28-9.09)*	2.90 (1.30-6.46)*	0.75 (0.27-2.08)	–	0.44 (0.17-1.15)	–
**Alcohol consumption**								
No	1	1	1	–	1	–	1	–
Yes	1.63 (1.23-2.17)**	1.16 (0.85–1.57)	1.18 (0.68-2.07)	–	0.90 (0.52-1.55)	–	1.32 (0.67-2.61)	–
**Age (years)**								
15-24	1	1	1	–	1	1	1	1
25-34	4.31 (2.76-6.73)**	4.08 (2.40-6.94)**	1.24 (0.66-2.33)	–	1.07 (0.62-1.84)	0.87 (0.48–1.58)	1.31 (0.98 - 1.74)	1.08 (0.79–1.48)
35-59	6.15 (4.03-9.39)**	5.61 (3.15-9.96)**	1.49 (0.84-2.66)	–	4.56 (2.87-7.25)**	2.83 (1.50–5.35)**	3.24 (2.54 - 4.13)*	2.90 (2.13–3.95)*
**Residence**								
Urban	0.83 (0.59-1.17)	–	1.51 (0.98-2.32)	–	0.32 (0.19-0.55)**	0.46 (0.27–0.79)**	1.05 (0.88-1.27)	**–**
Rural	1	1	1	1	1	1	1	–
**Region/Province**								
Coast	1	1	1	1	1	1	1	1
Northeastern	0.04 (0.01-0.32)**	0.16 (0.02-1.31)	0.33 (0.07-1.48)	1.46 (0.28–7.49)	0.00 (0.00-0.00)	0.00 (0.00–0.00)	0.45 (0.27-0.73)*	0.90 (0.29–2.74)
Eastern	0.73 (0.40-1.31)	0.76 (0.32-1.85)	1.52 (0.76-3.04)	1.67 (0.66–4.22)	3.04 (1.53-6.05)*	1.15 (0.38–3.54)	1.21 (0.89-1.65)	1.33 (0.71–2.51)
Central	1.01 (0.52-1.97)	1.08 (0.35-3.34)	3.30 (1.51-7.24)**	1.93 (0.63–5.91)	1.56 (0.80-3.06)	1.16 (0.35–3.89)	1.36 (0.96-1.92)	1.21 (0.58–2.54)
Rift Valley	2.20 (1.35-3.60)**	2.74 (1.14-6.58)**	3.06 (1.64-5.72)**	3.27 (1.29–8.28)**	1.12 (0.57-2.18)	1.24 (0.38–4.06)	0.29 (0.98-1.71)	1.58 (0.83–3.01)
Western	2.08 (1.15-3.79)**	2.94 (1.14-7.58)**	5.72 (3.00-10.90)**	9.23 (3.47–24.52)**	0.86 (0.38-1.98)	0.84 (0.22–3.11)	1.47 (1.08-2.01)*	2.44 (1.25–4.75)**
Nyanza	1.07 (0.60-1.91)	1.34 (0.46-3.87)	2.21 (1.08-4.55)**	4.63 (1.29–16.66)**	0.69 (0.31-1.54)	0.31 (0.05–1.85)	1.06 (0.79 - 1.42)	1.29 (0.57–2.90)
Nairobi	1.44 (0.64-3.24)	1.76 (0.61–5.06)	5.21 (2.11-12.86)**	4.12 (1.48–11.48)**	0.32 (0.04-2.44)	0.49 (0.06–3.90)	1.89 (1.29 - 2.79)*	2.25 (1.09–4.63)**
**Ethnicity**								
Embu	0.49 (0.15-1.59)	0.62 (0.16–2.30)	0.41 (0.13-1.36)	0.57 (0.14–2.33)	1.26 (0.49-3.27)	0.79 (0.25–2.52)	0.80 (0.49 - 1.31)	0.66 (0.34–1.26)
Kalenjin	**1.70 (1.09-2.66**)**	1.10 (0.61–1.97)	0.57 (0.31-1.07)	0.54 (0.26–1.12)	0.48 (0.26-0.92)*	0.38 (0.16–0.90)*	0.76 (0.59-0.98)**	0.78 (0.55–1.10)
Kamba	0.75 (0.35-1.63)	1.05 (0.33–3.36)	0.37 (0.13-1.08)	0.46 (0.10–2.02)	0.29 (0.12-0.67)*	0.32 (0.11–0.90)*	0.36 (0.25-0.53)*	0.36 (0.21–0.63)**
Kikuyu	1	1	1	1	1	1	1	1
Kisii	0.74 (0.21-2.59)	0.77 (0.18–3.30)	**0.10 (0.03-0.31)**	0.08 (0.02–0.29)*	0.16 (0.04-0.66)*	0.34 (0.08–1.54)	0.47 (0.24-0.93)**	0.47 (0.21–1.09)
Luhya	1.29 (0.80-2.10)	0.79 (0.39–1.59)	0.81 (0.46-1.43)	0.38 (0.17–0.87)*	0.51 (0.28-0.93)**	0.59 (0.26–1.34)	0.73 (0.57-0.94)**	0.52 (0.35–0.77)**
Luo	0.99 (0.54-1.81)	0.88 (0.35–2.24)	0.62 (0.27-1.43)	0.33 (0.08–1.33)	0.67 (0.28-1.60)	1.61 (0.40–6.44)	0.92 (0.66-0.28)**	0.72 (0.40–1.32)
Maasai	1.40 (0.70-2.81)	1.13 (0.51–2.48)	0.32 (0.05-1.95)	0.37 (0.06–2.36)	1.46 (0.52-4.04)	1.30 (0.42–4.04)	0.60 (0.33-1.09)**	0.71 (0.38–1.32)
Meru	0.75 (0.37-1.52)	0.95 (0.37–2.41)	0.48 (0.22-1.05)	0.74 (0.27–1.99)	5.41 (3.16-9.24)*	3.41 (1.73–6.72)*	1.57 (1.11-2.22)**	1.45 (0.91–2.30)
Mijikenda/Swahili	0.80 (0.42-1.53)	0.77 (0.27–2.22)	0.24 (0.11-0.53)*	0.78 (0.25–2.40)	0.80 (0.36-1.75)	0.74 (0.19–2.94)	0.61 (0.42-0.91)**	0.85 (0.40–1.80)
Taita/Taveta	0.69 (0.19-2.49)	0.79 (0.18-3.53)	0.00 (0.00-0.00)	0.00 (0.00–0.00)	0.63 (0.14-2.80)	0.76 (0.13–4.44)	1.28 (0.42-3.86)	1.57 (0.32–7.67)
Somali	0.08 (0.02-0.27)**	0.10 (0.02-0.47)*	0.08 (0.02-0.28)*	0.13 (0.03–0.59)*	0.00 (0.00-0.00)	0.00 (0.00-0.00)	0.35 (0.22-0.57)**	0.41 (0.13–1.31)
Other	0.68 (0.35-1.33)	0.46 (0.22-0.96)*	0.15 (0.06-0.38)*	0.12 (0.04–0.35)*	0.45 (0.24-0.87)*	0.45 (0.21–0.97)*	0.70 (0.46-1.05)	0.61 (0.36–1.02)
**Education**								
None/primary	1	1	1	1	1	1	1	1
Secondary	0.56 (0.41-0.74)**	0.92 (0.64–1.30)	1.17 (0.70-1.96)	1.06 (0.67–1.66)	0.35 (0.24-0.52)**	0.65 (0.43–0.99)*	0.80 (0.67-0.97)**	1.03 (0.85–1.25)
Tertiary	1.08 (0.71-1.64)	1.57 (0.99–2.50)	2.15 (1.32-3.52)**	1.52 (0.83–2.81)	0.56 (0.32-0.99)**	1.30 (0.74–2.29)	1.37 (1.09-1.73)**	1.56 (1.20–2.03)**
**Religion**								
Christians	1	1	1	–	1	1	1	1
Muslims	1.05 (0.63-1.74)	3.16 (1.66-6.02)**	0.70 (0.38-1.27)	–	0.65 (0.29-1.46)	1.33 (0.56–3.17)	0.87 (0.67-1.13)	1.48 (1.01–2.15)*
Others	1.52 (1.03-2.25)***	1.52 (1.01-2.26)**	1.33 (0.54-3.26)	–	3.02 (1.66-5.50)**	2.06 (1.20–3.54)*	1.47 (1.03-2.09)**	1.55 (1.03–2.31)*
**Marital status**								
Unmarried (single/divorced/widowed/separated)	1	1	1	–	1	1	**1**	1
Married/Cohabiting	1.91 (1.39-2.61)**	0.77 (0.51-1.16)	0.92 (0.59-1.44)	–	1.93 (1.25-2.99)**	1.09 (0.66–1.81)	1.83 (1.52-1.21)**	0.99 (0.79–1.24)
**Working status/occupation**								
Not Working	1	1	1	–	1	–	1	1
Working	2.96 (1.87-4.70)**	1.31 (0.79-2.17)	0.99 (0.50-1.97)	–	1.43 (0.92-2.23)	–	1.67 (1.25-2.22)**	0.98 (0.71–1.35)
**Wealth index**								
Poorest	1	–	1	1	1	–	1	1
Poorer	1.15 (0.76-1.73)	–	1.01 (0.56-1.85)	0.81 (0.44–1.50)	1.59 (0.88-2.88)	–	1.10 (0.86 - 1.40)	1.04 (0.80–1.34)
Middle	0.99 (0.65-1.49)	–	0.95 (0.54-1.68)	0.77 (0.42–1.41)	0.99 (0.56-1.76)	–	1.01 (0.81- 1.27)	1.01 (0.78–1.31)
Richer	0.96 (0.60-1.54)	–	0.79 (0.44-1.42)	0.64 (0.33–1.26)	0.71 (0.39-1.26)	–	1.15 (0.88-1.51)	1.13 (0.86–1.49)
Richest	0.82 (0.48-1.41)	–	2.60 (1.46-4.64)**	1.93 (1.07–3.50)*	0.58 (0.27-1.27)	–	1.57 (1.22-2.03)**	1.32 (0.92–1.90)
**Household number**								
≤4	1	–	1	–	1	–	1	–
≥5	1.31 (0.74-2.33)	–	0.84 (0.33-2.12)	–	1.05 (0.43-2.59)	–	1.24 (0.87-1.77)	–
**Health status**								
Bad	1		1	1	1	1	1	1
Moderate	0.38 (0.21-0.69)**	0.47 (0.25-0.89)*	0.29 (0.13-0.61)**	0.26 (0.12–0.59)**	0.41 (0.21-0.79)**	0.55 (0.25–1.23)	0.58 (0.40-0.85)*	0.63 (0.41–0.97)*
Good	0.14 (0.08-0.24)**	0.19 (0.10-0.34) *	0.15 (0.07-0.30)**	0.13 (0.07–0.27)**	0.12 (0.06-0.23)**	0.18 (0.09–0.39)**	0.20 (0.14-0.28)*	0.22 (0.14–0.34)*
**Newspaper**								
No	1	–	1	–	1	–	1	–
Yes	0.86 (0.62-1.18)	–	1.32 (0.86-2.03)	–	0.72 (0.48-1.07)	–	1.12 (0.95-1.32)	–
**TV**								
No	1	–	1	–	1	–	1	–
Yes	0.79 (0.55-1.14)	–	1.23 (0.75-2.02)	–	0.95 (0.62-1.47)	–	1.06 (0.88-1.29)	–
**Radio**								–
No	1	–	1	1	1	–	1	–
Yes	1.07 (0.67-1.71)	–	0.56 (0.31-1.00)*	1.79 (1.02-3.13)*	1.01 (0.59-1.73)	–	0.93 (0.70-1.22)	–
**Internet use**								–
No	1	1	1	1	1	1	1	–
Yes	0.72 (0.54-0.95)*	0.70 (0.49–1.00)	1.52 (1.02-2.25)*	1.09 (0.70–1.68)	0.51 (0.35-0.74)**	0.89 (0.60–1.33)	1.03 (0.88-1.22)	–
**Lifestyle Behavioural factors**								
**Tobacco use**								–
No	1	1	1	–	1	1	1	1
Yes	1.95 (1.39-2.73)**	1.14 (0.74–1.76)	1.11 (0.71-1.74)	–	2.97 (2.02-4.37)**	1.15 (0.74–1.81)	1.53 (1.26-1.86)*	1.05 (0.81–1.36)
**Number of hours per day seated/sedentary**		–						
Low risk (≤8 hours)/non-sedentary life	1	–	1	–	1	–	1	–
High risk (>8 hours)/sedentary life	0.90 (0.50-1.64)	–	0.70 (0.28-1.73)	–	1.18 (0.46-3.05)	–	1.15 (0.74-1.78)	–
**Alcohol consumption**						–		
No	1	1	1	–	1	–	1	1
Yes	1.73 (1.28-2.35)**	1.17 (0.83–1.64)	1.37 (0.87-2.17)	–	1.24 (0.82-1.88)	–	1.40 (1.16-1.69)*	1.03 (0.81–1.32)

Note. OR = unadjusted odd ratios, aOR = adjusted odds ratio, * = significant at p < 0.05, ** = significant at p < 0.001, *** = significant at p < 0.1, and – = not evaluated in that model.

**Table 4:** Factors associated with having multiple NCDs (multi-morbidity) among men in Kenya.

	Multiple NCDs (≥2)/multi-morbidity			
Variable (N = 14,439)	*n*	Yes, %	uORs (95 CI)	*aORs (95 CI)*
**Age (years)**				
15-24	5577	0.19	1	1
25-34	4049	0.47	3.54 (1.55-8.07)*	2.67 (1.00–7.15)**
35-59	4813	1.27	8.17 (3.72-17.96)*	7.17 (2.75–18.69)**
**Residence**				
Urban	5610	0.79	1.11 (0.76-1.62)	–
Rural	8829	1.13	1	–
**Region/Province**				
Coast	1341	0.14	1	1
Northeastern	273	0.01	0.32 (0.10-1.00)	2.34 (0.75–7.31)
Eastern	2123	0.18	0.86 (0.47-1.58)	0.70 (0.29–1.68)
Central	1945	0.26	1.35 (0.72-2.53)	1.56 (0.57–4.26)
Rift Valley	3808	0.40	1.05 (0.63-1.74)	1.76 (0.82–3.80)
Western	1499	0.29	1.96 (1.12-3.43)**	3.87 (1.70–8.84)*
Nyanza	1610	0.23	1.45 (0.85-2.48)	1.76 (0.68–4.53)
Nairobi	1841	0.41	2.25 (1.09-4.65)**	2.39 (1.00–5.69)*
**Ethnicity**				
Embu	186	0.02	0.77 (0.27-2.16)	0.93 (0.26–3.32)
Kalenjin	1823	0.14	0.54 (0.30-0.97) *	0.63 (0.29–1.38)
Kamba	1660	0.19	0.76 (0.35-1.65)	1.18 (0.39–3.51)
Kikuyu	2521	0.37	1	1
Kisii	901	0.04	0.31 (0.12-0.78) *	0.32 (0.11–0.95)**
Luhya	2424	0.35	1.00 (0.60-1.65)	0.62 (0.29–1.30)
Luo	1591	0.36	1.56 (0.85-2.85)	1.12 (0.37–3.38)
Maasai	322	0.04	0.94 (0.40-2.20)	1.58 (0.59–4.25)
Meru	717	0.16	1.60 (0.63-4.07)	2.73 (0.93–8.01)
Mijikenda/Swahili	798	0.10	0.84 (0.43-1.62)	2.11 (0.73–6.09)
Taita/Taveta	136	0.01	0.69 (0.20-2.31)	0.95 (0.24–3.75)
Somali	294	0.01	0.20 (0.07-0.63)*	0.27 (0.07–1.01)
Other	1065	0.14	0.87 (0.30-2.49)	0.95 (0.24–3.69)a
**Education**				
None/primary	5657	0.70	1	1
Secondary	5842	0.60	0.83 (0.56-1.24)	1.28 (0.87–1.89)
Tertiary	2940	0.63	1.74 (1.12-2.70)*	2.00 (1.26–3.18)*
**Religion**				
Christians	12347	1.73	1	–
Muslims	1042	0.11	0.73 (0.41-1.29)	–
Others	1050	0.09	0.63 (0.34-1.18)	–
**Marital status**				
Unmarried (single/divorced/widowed/separated)	7490	0.61	1	1
Married/Cohabiting	6949	1.31	2.34 (1.57-3.48)**	0.77 (0.46–1.28)
**Working status/occupation**				
Not Working	3157	0.14	1	1
Working	11282	1.79	3.63 (1.97-6.66)**	1.65 (0.72–3.80)
**Wealth index**				
Poorest	2175	0.22	1	1
Poorer	2762	0.35	1.27 (0.76-2.13)	1.17 (0.68–2.01)
Middle	2929	0.34	1.16 (0.68-1.96)	1.18 (0.67–2.06)
Richer	3493	0.40	1.14 (0.64-2.03)	1.07 (0.55–2.09)
Richest	3079	0.61	1.97 (1.11-3.50)***	1.51 (0.81–2.84)
**Household number**				
≤4	714	0.11	1	–
≥5	13735	1.82	0.86 (0.42-1.74)	–
**Health status**				
Bad	186	0.16	1	1
Moderate	2110	0.76	0.09 (0.05-0.16)*	0.35 (0.19–0.66)**
Good	12143	1.01	0.40 (0.22-0.75)*	0.09 (0.05–0.17)**
**Newspaper**				
No	8747	1.16	1	–
Yes	5692	0.77	1.02 (0.70-1.49)	–
**TV**				–
No	2873	0.38	1	–
Yes	11566	1.55	1.01 (0.68-1.51)	–
**Radio**				–
No	1817	0.23	1	–
Yes	12622	1.69	1.05 (0.61-1.80)	–
**Internet use**				–
No	5955	0.69	1	–
Yes	8484	1.24	1.27 (0.92-1.76)	–
Lifestyle Behavioral factors				
**Tobacco use**				
No	12517	1.56	1	1
Yes	1922	0.37	1.54 (1.05-2.26)*	0.93 (0.57–1.52)
**Number of hours per day seated/sedentary**				
Low risk (≤8 hours)/non-sedentary life	13692	1.78	1	–
High risk (>8 hours)/sedentary life	747	0.15	1.53 (0.60-3.91)	–
**Alcohol consumption**				
No	10600	1.20	1	1
Yes	3839	0.73	1.69 (1.18-2.43)**	1.09 (0.70–1.68)

Note. OR = unadjusted odds ratios; aOR = adjusted odds ratio; * = significant at p < 0.05, ** = significant at p < 0.001; *** = significant at p < 0.1; – = not evaluated in that model; n = row total; % = percentage of the total sample size when all the row totals are added up.

The findings of this study also indicated men from other regions of Kenya, when compared to those from the coastal region, were more likely to have heart disease (e.g., from the Northeastern (aOR 6.53 (95%CI:1.11–38.46), western (aOR 17.72 (95%CI:1.74–180.26)), Nyanza (aOR 13.97 (95%CI:1.12–174.52) and Nairobi (aOR 31.49 (95%CI:3.26–303.97), lung disease (e.g., Western = aOR 3.22 (95%CI:1.16–8.96), experience depression (e.g., Rift valley (aOR 2.20 (95%CI:1.35–3.60) and western (aOR 2.08 (95%CI:1.15–3.79), have arthritis (3.04 (95%CI:1.53–6.05), have at least one NCD (e.g., western (aOR 2.44 (95%CI:1.25–4.75), and multiple NCDs (e.g., western province =(aOR 3.87 (95%CI:1.70–8.84). Additionally, those from the Rift Valley (aOR 3.27 (95%CI:1.29–8.28), Western (aOR 9.23 (95%CI:3.47–24.52), Nyanza (aOR 4.63 (95%CI: 95%CI:1.29–16.66), and Nairobi (aOR 4.12 (95%CI:1.48–11.48) provinces of Kenya, when compared with those from the coastal region, were more likely to experience anxiety. However, those from the Northeastern province of Kenya, when compared with those from the coastal region, were less likely to experience depression (0.04 (95%CI:0.01–0.32).

We found that experiencing NCDs also varied across tribes. Compared with the Kikuyu tribe, other tribes of Kenya had higher odds of having heart disease (e.g., Taita/Taveta = aOR 23.63 (95%CI:1.18–471.56), and arthritis (e.g., Meru tribe = aOR 3.41 (95%CI:1.73–6.72). However, some ethnic tribes had lower odds of experiencing heart disease (e.g., Kisii tribe = aOR 0.07 (95%CI: 0.01–0.59), anxiety (e.g., Luhya = aOR 0.51 (95%CI:0.28–0.93), depression (e.g., Somali = aOR 0.10 (95%CI:0.02–0.47), arthritis (e.g., Kamba = aOR 0.32 (95%CI:0.11–0.90), hypertension (e.g., Kalenjin (aOR 0.59 (95%CI:0.36–0.95), having at least one NCD (e.g., Luhya (aOR 0.52 (95%CI:0.35–0.77), and multiple NCDs (Kisii = aOR 0.32 (95%CI:0.11–0.95).

Participants who completed secondary or tertiary education compared with those who had completed primary, or no education, had higher odds of having hypertension (aOR 1.67 (95%CI:1.11–2.51), lung disease (aOR 2.67 (95%CI:1.43–5.00), diabetes mellitus (aOR 2.11 (95%CI:1.15–3.87), at least one NCD (aOR 1.56 (95%CI:1.20–2.03), and multiple NCDs (aOR 2.00 (95%CI:1.26–3.18). However, completing secondary education was associated with lower odds of experiencing arthritis when compared with completing primary or no education (aOR 0.65 (95%CI: 0.43–0.99).

We found that men who perceived themselves to be in moderate to good health compared with those that thought their health status was bad had lower odds of having hypertension (aOR 0.22 (95%CI: 0.12–0.38), lung disease (aOR 0.29 (95%CI: 0.10–0.87), diabetes (aOR 0.11 (95%CI: 0.04–0.29), heart disease (aOR 0.19 (95%CI: 0.07–0.54), arthritis (aOR 0.18 (95%CI: 0.09–0.39), experiencing anxiety (aOR 0.13 (95%CI: 0.07–0.27), having at least one NCD (e.g., moderate = aOR 0.63 (95%CI: 0.41–0.97) and multiple NCDs (e.g., good health conditions = aOR 0.09 (95%CI: 0.05–0.17). We also found that men from the middle (aOR 3.09 (95%CI: 1.24–7.67), richer (aOR 3.32 (95%CI: 1.31–8.40) and richest (aOR 6.95 (95%CI: 2.76–17.55) indices when compared with those from the poorest wealth index, had a higher likelihood of experiencing diabetes mellitus. Likewise, men from the richest quintile, when compared with those from the poorest quintile, were more likely to experience anxiety (aOR 1.93 (95%CI: 1.07–3.50). Similarly, participants who lived a sedentary life (sat for > 8 hours per day) compared with those who did not (≤ 8 hours per day), had higher odds of having diabetes (aOR 2.90 (95%CI: 1.30–6.46).

This study found that men subscribing to the other faith in Kenya, compared with the Christians, had higher odds of having arthritis (aOR 2.06 (1.20–3.54), experiencing depression (e.g., Muslim faith (aOR 3.16 (95%CI: 1.66–6.02), and having at least one NCD (e.g., other faith (aOR 1.55 (1.03–2.31). This study also found that living in urban settings was associated with less odds of having arthritis (aOR 0.46 (0.27–0.79) when compared with living in rural settings.

Finally, men who had access to radio, compared with those who did not, had higher odds of experiencing anxiety (aOR 1.79(95%CI:1.02–3.13). Whereas those who had access to all mass media channels, compared with those who did not, had lower odds of experiencing lung disease (aOR 0.19 (95%CI:0.08–0.47).

## Discussion

The study aimed at determining the prevalence and associated factors of non-communicable diseases among men in Kenya. Although many of the identified risk factors for NCDs have been reported in prior studies, this analysis contributes to the literature by focusing specifically on men aged 15–54 years using nationally representative data. Young and middle-aged men remain an understudied population in NCD epidemiology, despite representing a key demographic for early prevention. Our findings, therefore, provide critical insights into the early distribution of NCDs and their associated risk factors in this age group, which can inform age and gender-responsive prevention strategies. Additionally, the use of complex samples methods allows for more accurate population-level estimates within this demographic, filling an important evidence gap in the region.

The study findings revealed that the overall prevalence of NCDs among men in Kenya was 9.4%, with hypertension, depression, and anxiety being the most common conditions. These findings align with previous studies in other countries, such as Lesotho and Nepal, which showed hypertension to be the leading NCD among men [13,21]. Although there is a dearth of published data of the overall prevalence in other East African countries, the overall prevalence is lower than that in India, of 12.4% and 15.9% of Kenyan women with at least one NCD [14,22]. This variation in the prevalence of NCDs across countries could be due to differences in socioeconomic factors, healthcare access, lifestyle habits, and the age distribution of the populations [23].

In this study, hypertension emerged as the most prevalent NCD, with a significant proportion of the population affected being 3.5%, which is lower than the 21% recorded among seven East African countries [24] and the 41.4% prevalence of the 2020 Global Epidemiology Study [25]. Similarly, the very low prevalence of cancer (0.1%) in this study contrasts with the increasing global burden of cancer, especially in low and middle-income countries [26]. However, these comparisons should be interpreted cautiously because our study population included only men aged 15–54 years. Hypertension, as well as cancer, exhibits a strong age gradient, with prevalence rising sharply in older adults. Therefore, the lower prevalence observed in our sample likely reflects the younger age structure of the study population rather than true differences in underlying disease burden. This discrepancy may also reflect underreporting, limited early detection capacity, or screening within the primary care setting, particularly among men. Therefore, these findings suggest a critical need for concerted and sustained efforts to enhance NCD screening and surveillance at the community level among men in Kenya and other sub-Saharan African countries.

NCD multimorbidity poses substantial challenges, including increased healthcare costs and complex disease management, yet its prevalence remains insufficiently documented in sub-Saharan Africa. In this study, the majority of the participants had diabetes and hypertension, and this co-existence is supported by literature, as both conditions share several risk factors related to their development, such as obesity, poor diet, physical inactivity, and genetic predisposition [27]. The co-occurrence of diabetes and hypertension significantly increases the risk of cardiovascular complications, including stroke, heart failure, and kidney disease [28]. Accordingly, stakeholders such as the Kenyan Ministry of Health may need to strengthen research and public awareness on NCD multimorbidity, promote early screening and treatment, and support lifestyle-modification interventions to prevent its onset and progression.

Our findings indicate a co-occurrence of mental and somatic NCDs, with the most common combination being hypertension and depression, followed by hypertension and anxiety. This is consistent with different studies, which have reported that depression and anxiety can negatively impact blood pressure regulation, resulting in hypertension [29,30]. The frequent co-occurrence of mental health conditions with hypertension highlights the interplay between physical and psychological health [29]. Furthermore, the co‑occurrence of arthritis and depression observed in our study aligns with existing literature documenting a significant association between these two conditions [31]. Chronic pain and mobility limitations associated with arthritis often lead to reduced quality of life, social isolation, and emotional distress, making depression a common comorbidity [32]. The observed multimorbidity patterns in this study may point to the inadequacy of vertical disease programs and demonstrate the necessity for integrated, person-centered NCD care models. Therefore, the governments of Kenya and SSA countries may need to prioritize and integrate mental health screening and support into chronic disease management.

This study also identified several factors associated with the prevalence of NCDs, including increasing age, region, residence, ethnicity, education level, perceived health status, wealth index, religion, media access, and living a sedentary lifestyle. Age was statistically significantly associated with NCDs among men, where men aged 25 years and older were more likely to experience at least one or multiple NCDs, including hypertension, diabetes, depression, and arthritis, compared with the younger participants. This concurs with the existing literature in India and Ghana, which emphasizes that aging is a key risk factor for NCDs [33,34]. However, the finding is inconsistent with a study in Burkina Faso that reported NCDs tend to be higher in younger populations due to lifestyle factors such as increasing urbanization, sedentary behavior, and dietary shifts towards processed foods [35]. The aging process involves major physiological changes like reduced organ function and inflammation, which makes an individual prone to chronic illness [36]. Therefore, the Kenyan and other SSA health systems may need to prepare for rising long-term care needs as their population ages, implement preventive programs among younger men, and age-targeted screening programs. A lot of emphasis should be put on the health education of men regardless of their age, about healthy living, physical exercises, and routine medical checkups.

This study found that region was statistically significantly associated with NCDs among men. Men from Western, Rift Valley, Nairobi, and Nyanza provinces were more likely to experience heart disease, lung disease, depression, anxiety, have at least one NCD, and multiple NCDs compared with those from the coastal region of Kenya. The study finding is consistent with findings from a study in Kenya, which reported that regional differences influence the prevalence of NCDs [8,37]. Urban areas like Nairobi and the Coast were found to have high NCD rates due to sedentary lifestyles, unhealthy diets, and substance use, especially in informal settlements. Although NCDs are often associated with urbanization, our findings showing elevated NCD risk among men in less urbanized regions such as Nyanza, Northeastern, and Rift Valley are consistent with emerging evidence that rural populations in sub‑Saharan Africa also experience substantial NCD risk. Studies demonstrate that rural residents may exhibit significant exposure to risk factors, including tobacco and alcohol use, and face structural barriers such as limited access to screening and essential NCD services, contributing to delayed diagnosis and poorer outcomes [38–40]. Even in rural areas, the growing use of processed foods is increasing NCD risk [5]. Therefore, this study findings may demonstrate the presence of regional inequities in detection, diagnosis, and care, which may require the government of Kenya to develop and implement region-specific public health education campaigns and policies, such as the rural tobacco tax that addressed the root causes of NCDs in high-risk regions of India [41].

Tribe was statistically significantly associated with NCDs among men, as men from the Meru, Kamba, Taita/Taveta, Luhya, and Embu tribes had higher odds of experiencing NCDs like arthritis and heart disease, compared with the Kikuyu tribe. However, men from some ethnic tribes had lower odds of experiencing hypertension, anxiety, depression, heart disease, having at least one NCD, and multiple NCDs compared with the Kikuyu tribe. The findings from this study regarding tribal differences in NCDs concur with a study in Kenya, which reported the rise in NCDs in certain tribes [8]. This was attributed to increased urbanization, the declining proportion of agricultural and forestry wage jobs, and unhealthy eating [8]. These ethnic differences may reflect genetic, cultural, occupational, lifestyle, or environmental factors that influence disease susceptibility [42]. Therefore, health workers, among other stakeholders, may need to address ethnic disparities in NCDs using culturally sensitive approaches, like designing NCD education campaigns in local languages [43].

Participants who completed secondary or tertiary education, compared with those who had completed primary or no education, had higher odds of having hypertension, diabetes mellitus, at least one NCD, and multiple NCDs, unlike experiencing arthritis. This finding concurs with previous research in India that showed a strong association between higher education levels and increased NCDs among men [44]. However, the findings are inconsistent with a study in China and a review, which reported lower level of education was highly associated with hypertension and diabetes mellitus [45,46]. Higher education in men may increase NCDs due to lifestyle changes, such as inactivity, which is associated with white-collar jobs [47]. On the other hand, educated individuals tend to have better access to healthcare, early screening services that may lead to high detection rates, and coping strategies. [48]. Individuals, regardless of their education status, may need to be encouraged by various health sector stakeholders to regularly screen for NCD risk factors. Educated and working men in Kenya and other SSA countries may need to recognize or modify their lifestyle NCD risk factors associated with their jobs, such as long hours of inactivity, poor dietary habits, and high stress.

We also found that men with a higher wealth index, when compared with those from the poorest wealth index, had higher odds of having diabetes mellitus and anxiety. The findings concur with a study in Bangladesh, which reported that diabetes risk was about 60% higher in adults from wealthy households [49]. Unfortunately, the study findings are contrary to a study in South Africa, which reported a high prevalence of diabetes among the poor [50]. Higher economic status can lead to sedentary lifestyles, stress, and unhealthy eating [51]. Likewise, contrary to our study findings, wealthier individuals experience less anxiety due to better access to healthcare, financial security, and lower stress levels, which satisfy their psychosocial needs [52]. Health workers should prioritize educating men in higher wealth brackets on the importance of maintaining a balanced diet and engaging in regular physical activity.

This study also found that participants from the urban setting, when compared with those from the rural setting, were less likely to have arthritis and at least one NCD. The findings concur with two studies, one in 47 counties of Kenya and another that utilized data from the 2022 KDHS, which reported a high prevalence of cardiovascular diseases in rural areas [14, 37]. However, the study findings contrast with a study in Belagavi, which reported that participants in urban areas had a high risk of NCDs and had a high rate of smoking [53]. Rural areas have limited access to health care diagnostics, better transport systems, and education about healthy living. Lack of diagnostics and medical professionals delays early detection and treatment. Additionally, poor health literacy and unhealthy behaviors exacerbate NCD risks [54]. Arthritis is less common in urban settings due to better healthcare access, healthier lifestyles, and more opportunities for physical activity [55]. On the other hand, rural men may experience occupational injuries related to excessive manual labor, leading to arthritis. Policy makers in Kenya and other SSA countries should focus on improving health awareness and early screening of arthritis in rural areas to ensure timely detection and management.

Men who had access to mass media compared with those who did not have access to it had lower odds of having lung disease and higher odds of having anxiety. The findings are consistent with different studies, which reported an association between access to radio and anxiety, and access to the internet with having at least one NCD [22,56,57]. Access to radio and the internet may contribute to anxiety and unhealthy behaviors due to information overload, exposure to distressing news, misinformation, and lifestyle influences from digital media [22]. Therefore, because access to mass media may be beneficial in reducing the risk of some NCDs and detrimental in others, further research is needed to explore how media exposure, media content, and health messaging may influence men’s exposure to NCDs in Kenya and other sub-Saharan countries.

Participants who lived a sedentary life compared with those who did not, had higher odds of having diabetes. The findings concur with a global study which reported that a sedentary lifestyle is associated with inactivity and poor eating habits that increase the risk of type 2 diabetes [58,59]. This emphasizes that a sedentary lifestyle is a significant risk factor for the development of diabetes, which is a growing global health concern. Individuals should be encouraged to make small, gradual changes to reduce sedentary time and incorporate physical activity into their daily routines [59]. As part of the public health initiatives aimed at reducing the incidence of diabetes, stakeholders may need to advocate and prioritize preventive community-level interventions that promote physical activity, such as wellness and sports programs.

Religion was significantly associated with NCDs. Men subscribing to the Muslim or other faith compared with the Christians had higher odds of having arthritis, depression, and at least one NCD. The findings concur with a study that reported a low rate of NCDs in Christian communities in the United States due to the counselling and coaching they receive to reduce NCDs [42]. Religious practices may influence health outcomes, as they often have specific health-related behaviors and social support networks that could either mitigate or exacerbate the risk of chronic diseases [60]. Further research is needed to explore how religious contexts may influence men’s exposure to NCDs in Kenya and other sub-Saharan countries. Additionally, faith communities and leaders may need to be involved in co-designing and implementing NCD interventions such as mental health messaging.

We found that men who perceived themselves to be in moderate to good health compared with those who thought their health status was bad had lower odds of having hypertension, lung disease, diabetes, heart disease, arthritis, anxiety, and having at least one NCD and multiple NCDs. Similar findings were observed in a study, which indicated that self-rated health is a strong predictor of physical health outcomes [61]. Improving self-perception of health could lead to better health behaviors and outcomes [62]. Therefore, as a low-cost intervention, governments in the SSA region may need to integrate mental health screening into routine health perception assessments to motivate proactive health management and encourage behavior change.

### Strengths and limitations

We used the most-recent data from the 2022 Kenya Demographic and Health survey, with a large sample size and standardized data collection protocols ensuring that the findings are robust, reflect current epidemiological patterns within the country, and provide evidence that can meaningfully inform health policy and targeted NCD prevention efforts. However, this study analysed all self‑reported, provider‑diagnosed non‑communicable diseases included in the 2022 KDHS men’s questionnaire, which were clinically heterogeneous and may differ in prevalence and symptom onset within the 15–54‑year age range. The fact that the study is based on retrospective information provided by the survey respondents, may have introduced recall, reporting (misclassification), and socio-desirability bias since it did not allow verification of clinical assessments or disease severity. Thus, our study outcomes are subject to under-diagnosis. Additionally, given the cross‑sectional design, the observed associations should not be interpreted as causal, nor can the study determine the underlying mechanisms linking sociodemographic factors to specific NCD outcomes. Secondly, due to a small sample size (n = 8) of those who self-reported to have cancer, further analysis of data related to cancer was not undertaken. Although our multivariable models adjusted for multiple structural, individual, behavioral, and community factors, the possibility of residual confounding cannot be excluded. The KDHS did not collect several important NCD risk determinants, including dietary composition, salt intake, cholesterol levels, genetic predisposition, environmental exposures (e.g., air pollution), detailed occupational risks, and stress levels. Additionally, some covariates such as sedentary behavior, and perceived health are self-reported and may be prone to misclassification. As a result, unmeasured or imperfectly measured confounders may have influenced the associations observed in this study.

## Conclusions

This study showed a relatively low prevalence of non-communicable diseases (NCDs) among men in Kenya, with hypertension, depression, and anxiety being the most common conditions. Factors such as age, region, residence, ethnicity, education level, health status, wealth index, religion, media access, and living a sedentary lifestyle were found to be associated with having/experiencing various NCDs, reflecting both individual and environmental influences. To reduce the burden of non-communicable diseases (NCDs) among men, several targeted and integrated strategies are recommended. The observed multimorbidity patterns in this study may point to the inadequacy of vertical disease programs and demonstrate the necessity for integrated, person-centered NCD care models. Older men may receive tailored health education, regular medical checkups, and physical activity promotion. Region-specific public health policies are needed to address localized risk factors, while culturally sensitive interventions may target ethnic disparities. Men regardless of their education and wealth status may need be encouraged to adopt healthier lifestyles and undergo routine screenings to mitigate risks linked to sedentary jobs and affluence. In rural areas, improving healthcare access and NCD awareness is essential. Further research is needed to explore how media exposure, media content, and health messaging may influence men’s exposure to NCDs in Kenya and other sub-Saharan countries. Promoting physical activity and reducing sedentary behavior are key to lowering hypertension and diabetes risk. Further research is needed to explore how religious contexts may influence men’s exposure to NCDs in Kenya and other sub-Saharan countries. Additionally, faith communities and leaders may need to be involved in co-designing and implementation of NCD interventions such as mental health messaging. Finally, as a low-cost intervention, governments in the SSA region may need to integrate mental health screening into routine including health perception assessments to motivate proactive health management and encourage behavior change.

## Supporting information

S1 Table Multicollinearity diagnostics using the Variance Inflation Factor (VIF) method. (DOCX)

S2 Table Strengthening the reporting of observational studies in epidemiology (STROBE) checklist. (DOC)
