# Supplementary material for: Prevalence and associated factors of non-communicable diseases among men: A cross-sectional analysis of Kenya Demographic and Health Survey 2022 data
Source: PLoS One. 2026 Jul 20;21(7):e0327266. doi: 10.1371/journal.pone.0327266 (PMC13384318; doi:10.1371/journal.pone.0327266)
Supplement: S1 Table — (DOCX) [file pone.0327266.s001.docx]

| **Predictors** | **Tolerance** | **VIF** |
| --- | --- | --- |
| Age (years) | 0.45 | 2.21 |
| Residence | 0.55 | 1.83 |
| Region/Province | 0.89 | 1.13 |
| Ethnicity | 0.95 | 1.05 |
| Education | 0.65 | 1.55 |
| Religion | 0.91 | 1.10 |
| Marital status | 0.51 | 1.97 |
| Working status/occupation | 0.73 | 1.37 |
| Wealth index | 0.42 | 2.40 |
| Household number | 1.00 | 1.00 |
| Health status | 0.96 | 1.04 |
| Newspapers | 0.85 | 1.18 |
| TV | 0.81 | 1.24 |
| Radio | 0.92 | 1.09 |
| Internet use | 0.62 | 1.61 |
| Tobacco Use | 0.78 | 1.28 |
| Number of hours per day seated/sedentary | 0.99 | 1.02 |
| Alcohol consumption | 0.84 | 1.19 |
| **MEAN** | **0.77** | **1.40** |

TABLE S1: Multicollinearity Diagnostics Using the Variance Inflation Factor (VIF) Method

Note—VIFs for all predictors were below the threshold of 3, indicating that multicollinearity was not problematic in our analyses.
