# Supplementary material for: Prevalence and associated factors of non-communicable diseases among men: A cross-sectional analysis of Kenya Demographic and Health Survey 2022 data
Source: PLoS One. 2026 Jul 20;21(7):e0327266. doi: 10.1371/journal.pone.0327266 (PMC13384318; doi:10.1371/journal.pone.0327266)
Supplement: Attachment — Submitted filename: Response to Reviewers.docx [file pone.0327266.s005.docx]

| **#** | **COMMENT** | **RESPONSE** | **PAGE/LINE No.** |
| --- | --- | --- | --- |
|  | **EDITOR COMMENTS** |  |  |
| 1 | Tighten up the research gap by highlighting the sex-specific rationale | Thank you very much for the comment. There are basically three reasons why we chose to do analysis of data related to men. First male-specific epidemiological data or analyses are scarce as mentioned by reviewer #3. This is partly because men are often treated as a homogenous and empowered population in global health and issues related to men are given less focus. second, a similar analysis had been conducted and published among women of reproductive age in Kenya (<https://doi.org/10.1136/bmjopen-2023-078666>). Therefore, we wanted to provide this evidence for better NCD policy formulation in Kenya. Thirdly, although we did not do a comparative analysis, we needed to explore whether factors that influenced NCD in women in Kenya as mentioned in literature were similar to those in men. These 3 reasons have been clarified in the introduction with following statement. “In this study, we focused exclusively on men for three reasons: (1) male‑specific epidemiological analyses remain limited in sub-Saharan african health literature, as men are often regarded as a homogenous and inherently advantaged group and therefore receive less analytical attention; (2) comparable analyses have already been conducted among Kenyan women of reproductive age (https://doi.org/10.1136/bmjopen-2023-078666). Generating parallel evidence for men is essential for balanced and comprehensive NCD policy formulation; and (3) examining men separately allows us to assess whether the prevalence and determinants of NCDs previously documented among women in Kenya are consistent with those observed in the male population.” | PG 4-5 LN 102-111 |
| 2 | Table 3 is compacted, which makes it quite difficult to comprehend. I suggest you revise it by focusing on the adjusted odds ratio and associated confidence intervals | Thank you very much for the suggestions. We had presented the statistics based on the available APA rules that adjusted odds ratio should be provided together with their corresponding crude and raw data. Nonetheless, we have provided a summarized table in the manuscript with crude and adjusted odds ratios with their associated confidence intervals alone. The rest of the variables have been deleted. | PG 18-25 |
| 3 | Revise your conclusion based on findings from your study to avoid establishing a causal relationship | Thank you very much for the comment or suggestions. We have revised the conclusions based on the reviewers comments both in the abstract and discussion section. | PG, 2-3, LN 49-61, 28-38 |
|  | **REVIEWER # 1** |  |  |
|  | The manuscript titled “Prevalence and associated factors of non-communicable diseases among men in Kenya: an analysis of the 2022 Kenya Demographic and Health Survey” is a well written article that explored the prevalence and social, demographic, behavioral factors of NCD in Kenya. The study was conducted on a larger sample size, using appropriate statistical methods making it relevant for public health interventions to tackle NCD. It also satisfies the PLOS ONE criteria for publication. However, there are some minor issues that need to be corrected and clarified. | Thank you very much for your compliment |  |
| 1 | Title The study was conducted among the Kenyan Male population. Please justify why the study excluded women and the relevance of this segmented study for the policy. | Thank you very much for the comment. There are basically three reasons why we chose to do analysis of data related to men. First male-specific epidemiological data or analyses are scarce as mentioned by reviewer #3. This is partly because men are often treated as a homogenous and empowered population in global health and issues related to men are given less focus. second, a similar analysis had been conducted and published among women of reproductive age in Kenya (<https://doi.org/10.1136/bmjopen-2023-078666>). Therefore, we wanted to provide this evidence for better NCD policy formulation in Kenya. Thirdly, although we did not do a comparative analysis, we needed to explore whether factors that influenced NCD in women in Kenya as mentioned in literature were similar to those in men. These 3 reasons have been clarified in the introduction with following statement. “In this study, we focused exclusively on men for three reasons: (1) male‑specific epidemiological analyses remain limited in sub-Saharan african health literature, as men are often regarded as a homogenous and inherently advantaged group and therefore receive less analytical attention; (2) comparable analyses have already been conducted among Kenyan women of reproductive age (https://doi.org/10.1136/bmjopen-2023-078666). Generating parallel evidence for men is essential for balanced and comprehensive NCD policy formulation; and (3) examining men separately allows us to assess whether the prevalence and determinants of NCDs previously documented among women in Kenya are consistent with those observed in the male population.” | PG 4-5 LN 102-111 |
| 2. | Page 2: line # 49-50: This study has exclusively assessed the prevalence and associated factors of NCD among Men. And the authors concluded that “The overall prevalence of NCDs among men is relatively lower than that of Kenyan women.” How this conclusion is possible without studying the female population? The conclusion part of an article should be based on the findings of its own result | Thank you for this insightful comment. We agree that our study assessed only men, and therefore direct comparisons with the prevalence of NCDs among Kenyan women cannot be presented as findings of this study. Our intention was to provide contextual information from previous national surveys; however, we acknowledge that this statement was inappropriate for the conclusion section.  We have revised the conclusion to focus strictly on the findings derived from our data. The comparative statement regarding women has been removed from the conclusion and, where necessary, clarified and properly referenced in the discussion section to provide external context.  We have edited the conclusion and now it reads: “*The overall prevalence of NCDs among men is relatively low based on available national data”*  *“”* | PG 2LN 49-50 |
| 3 | Result  Page 9, Table 1: The table title doesn’t fully explain the content. It says “socio-demographic characteristics …” But it has also behavioral and clinical variables. Consider revision. | Thank you very much for the suggestions. We agree that the original title of Table 1 did not fully reflect the range of variables included. We have revised the table title to more accurately capture the Socio-determinants of health model concepts. The table title now reads: *“Individual, behavioral, community, and structural characteristics of the study participants and their NCD status.”* | PG 11 LN 238-239 |
| 4 | Page 9, Table 1: The table is too long to the reader. Consider splitting. | Thank you very much for the comment. While we acknowledge that the table extends across two pages, dividing it into separate tables would result in unnecessary fragmentation and conflict with the journal’s guidelines on the number of permissible tables and figures. Instead, we have added a clear header on each page to improve readability. We therefore respectfully request to retain the table in its current format. | PG 11-12 |
| 5 | Page 11, Table 2: The total number of participants living with ≥2 NCDs is reported as n = 14174 (1.9%). However, if 14174 of them have ≥2 NCDs, it will be it will be 98.1%. Please revise the number. | Thank you for pointing out this inconsistency in Table 2. The value reported for participants living with ≥2 NCDs was indeed incorrect due to a typographical/data entry error. We have carefully reviewed the dataset and corrected both the frequency and percentage in the revised table to accurately reflect the number of participants with ≥2 NCDs. The current figure is 245 and not 14174. | PG 13 |
| 6 | Page 12, line # 225: Add space between aOR and the number. | Thank you very much for the suggestions. We have added the space | PG 14 LN 262 |
| 7 | Page 14, Line # 277: The study found that religion was among one of the statistically significant variables. Since religion can be sensitive issue in many contexts, please proof that it will not be point of dispute | Thank you for this important comment. We acknowledge that religion is a sensitive variable in many contexts. In this study, religion was included solely as a socio-demographic characteristic routinely captured in national surveys, with no intention to imply value judgments or causal interpretations regarding any religious group. The observed associations are strictly statistical and should be interpreted within the broader social and cultural context. We have clarified this in the manuscript and emphasized that the findings are not meant to stigmatize or single out any religious community. We also compared our finding to similar studies elsewhere that found a similar association and provided the most plausible reasons as to why such an association may exist. | PG 35-36 LN 507-517 |
|  | **REVIEWER # 2** |  |  |
| 1 | Line 51: Given the cross-sectional design, recommendations should be framed more cautiously, avoiding causal language (e.g., implying that doing X will reduce Y). This also applies to the discussion and conclusion. | Thank you for this valuable comment. We agree that, given the cross‑sectional nature of the study, recommendations and conclusions should avoid implying causality. We have revised the statements to use more cautious and non‑causal language. The recommendations now emphasize that approaches such as health education, routine checkups, and physical activity promotion may be relevant considerations rather than definitive interventions that reduce NCDs.  The following statement has been added to the abstract. “*Although causal relationships cannot be inferred from this study, the findings suggest that initiatives such as tailored health education, regular medical checkups, and the promotion of physical activity may be relevant components of broader efforts to support men’s health in relation to non‑communicable diseases (NCDs). Region‑specific policies and culturally sensitive approaches may help address variations in risk factors and observed ethnic disparities. Encouraging healthier lifestyle practices among men of all backgrounds, and improving access to specialized healthcare in rural areas, may also be important considerations.* Further research is needed to explore how media exposure, media content, health messaging, and religious contexts may influence men’s exposure to NCDs in Kenya and other sub-Saharan countries.” | PG2-3 LN51-60 |
| 2 | Line 49: The current study does not support this conclusion. | Thank you for this insightful comment. We agree that our study assessed only men, and therefore direct comparisons with the prevalence of NCDs among Kenyan women cannot be presented as findings of this study. Our intention was to provide contextual information from previous national surveys; however, we acknowledge that this statement was inappropriate for the conclusion section.  We have revised the conclusion to focus strictly on the findings derived from our data. The comparative statement regarding women has been removed from the conclusion and, where necessary, clarified and properly referenced in the discussion section to provide external context.  We have edited the conclusion and now it reads: “*The overall prevalence of NCDs among men is relatively low based on available national data”“”* | PG 2LN 49-50 |
| 3 | Line 54: Although the findings indicate higher NCD prevalence among wealthier and more educated men, recommendations for healthy lifestyle promotion should apply broadly across socioeconomic groups. The recommendations should be framed more inclusively. | Thank you very much for the suggestion: We agree that recommendations related to healthy lifestyle promotion should be inclusive and not limited to specific socioeconomic groups. We have revised the statement to emphasize that health‑promoting behaviors are important for men across all socioeconomic backgrounds. The statement now reads: “*Encouraging healthier lifestyle practices among men of all backgrounds”* | PG 2 LN56-57 |
| 4 | Line 56: While the conclusions may be reasonable in a broader public health context, they extend beyond the findings of this study. The analysis does not examine media content, health messaging, or stigma. | Thank you for this comment. We agree that the original statement extended beyond the scope of our analysis, as the study did not assess media content, health messaging, or stigma. We have revised the text to ensure that our conclusions remain aligned with the data. The revised statement focuses only on the observed associations and avoids extrapolations that are not supported by the study findings. The following statement has been added *“Further research is needed to explore how media exposure, media content, health messaging, and religious contexts may influence one’s exposure to NCDs in Kenya and other SSA countries.””* | PG 2-3 LN 56-60 |
| 5 | Line 60: The introduction outlines the burden of NCDs, differences in life expectancy by sex, and patterns of risk behaviors. However, it does not clearly explain why the study focuses specifically on men. Clarifying the sex-specific rationale would strengthen the argument and make the study objective more convincing. | Thank you very much for the comment. There are basically three reasons why we chose to do analysis of data related to men. First male-specific epidemiological data or analyses are scarce as mentioned by reviewer #3. This is partly because men are often treated as a homogenous and empowered population in global health and issues related to men are given less focus. second, a similar analysis had been conducted and published among women of reproductive age in Kenya (<https://doi.org/10.1136/bmjopen-2023-078666>). Therefore, we wanted to provide this evidence for better NCD policy formulation in Kenya. Thirdly, although we did not do a comparative analysis, we needed to explore whether factors that influenced NCD in women in Kenya as mentioned in literature were similar to those in men. These 3 reasons have been clarified in the introduction with following statement. “In this study, we focused exclusively on men for three reasons: (1) male‑specific epidemiological analyses remain limited in sub-Saharan african health literature, as men are often regarded as a homogenous and inherently advantaged group and therefore receive less analytical attention; (2) comparable analyses have already been conducted among Kenyan women of reproductive age (https://doi.org/10.1136/bmjopen-2023-078666). Generating parallel evidence for men is essential for balanced and comprehensive NCD policy formulation; and (3) examining men separately allows us to assess whether the prevalence and determinants of NCDs previously documented among women in Kenya are consistent with those observed in the male population.” | PG 4-5 LN 102-111 |
| 6 | Line 115: Please clarify how the reported 98% response rate was calculated. According to the 2022 KDHS report, 16,552 men aged 15–54 were eligible for individual interviews, and 14,453 were successfully interviewed (response rate: 87%). Additionally, please specify the inclusion and exclusion criteria applied in the analysis. | Thank you for this important comment. We appreciate the clarification regarding the response rate. The originally reported figure of 98% was incorrect. As indicated in the 2022 KDHS report, a total of 16,552 men aged 15–54 were eligible for individual interviews, and 14,453 were successfully interviewed, resulting in an overall response rate of approximately 87% (14,453/16,552 × 100). We have corrected this in the revised manuscript. The following statement has been added: “*In the households selected for the male survey, a total of 16,552 men aged 15–54 were identified as eligible for individual interviews, of whom 14,453 completed the interview, corresponding to a response rate of 87%*.”  In addition, we have now clearly specified the inclusion and exclusion criteria. *Men were included in the analysis if they: (1) were aged 15–54 years; (2) were usual household members or present the night before the survey; and (3) completed the individual men’s full household questionnaire. Men were excluded if they: lacked information on main covariates required for multivariable analysis.* These criteria have been added to the Methods section for clarity. | PG 5 LN 125-131 |
| 7 | Line 124: The included NCDs are heterogeneous. It would be helpful to provide a clearer rationale to justify their inclusion, particularly given the 15–54 year age group. | Thank you for this insightful comment. We agree that the eight NCDs included in the analysis represent a heterogeneous group. Our selection was guided by the structure of the 2022 KDHS men’s questionnaire, which captures all physician‑ or provider‑diagnosed chronic conditions under a single standardized NCD module. To ensure completeness and comparability with DHS‑based analyses, we included all NCDs reported in the survey. Although some of these conditions such as cancer or heart disease are less common in the 15–54‑year age group, the KDHS collects these data intentionally to monitor early‑onset NCDs, which are increasingly relevant in the Kenyan context. For example, some chronic diseases, such as depression, anxiety, and hypertension, frequently appear before age 40. KDHS is not assuming that most men 15–54 will have cancer or heart disease. Rather, KDHS collects this information deliberately so Kenya can track NCDs at earlier ages, monitor trends over time, detect emerging risks, and guide long‑term health planning, prevention and policy. However, we also agree with you some elements of this section may constitute a study weakness and the following statement to the limitation section *“However, this study analysed all self‑reported, provider‑diagnosed non‑communicable diseases included in the 2022 KDHS men’s questionnaire which were clinically heterogeneous and may differ in prevalence and symptom onset within the 15–54‑year age range. The fact that the study is based on retrospective information provided by the survey respondents, may have introduced recall, reporting (misclassification), and socio-desirability bias since it did not allow verification of clinical assessments or disease severity. Thus, our study outcomes are subject to under-diagnosis..*” | PG 36 LN 531-536 |
| 8 | Line 164: “multivariate” should be replaced with “multivariable,” considering separate regression models were fitted for each NCD outcome rather than modeling multiple outcomes simultaneously. | Thank you for this observation. We agree with the reviewer that the term “multivariable” is more appropriate in this context, as our analysis involved fitting separate regression models for each NCD outcome, rather than modeling multiple outcomes simultaneously. In accordance with this suggestion, we have replaced “multivariate” with “multivariable” . | PG 8 LN 194 |
| 9 | Line 166: The term “simple multivariate logistic regression” is not standard. | Thank you very much for the comment. We agree with the reviewer that the phrase “simple multivariate logistic regression” is not appropriate terminology. Our intention was to describe a bivariable (or univariable) logistic regression, where each predictor was individually assessed for its association with the NCD outcomes before inclusion in the multivariable model. The statement has been adjusted to:  “*All variables with P-values less than 0.05 in the univariable complex samples logistic regression analysis,* *based on the Wald F test were included in the subsequent multivariable logistic regression model to determine factors associated with the prevalence of NCDs.*” | PG 8 LN 192-195 |
| 10 | Line 166: Were predictors retained based on an omnibus test (e.g., likelihood ratio or Wald test) | Thank you for this insightful comment. In this analysis, we used the SPSS Complex Samples module, which applies the Wald F statistic as its primary omnibus test for evaluating the significance of predictors while accounting for the complex survey design.  During Univariable analysis, variables with p < 0.05 based on the Wald F test were considered for inclusion in the multivariable model. All eligible variables were then entered simultaneously into a multivariable Complex Samples logistic regression model, where the Wald F test continued to serve as the omnibus measure of statistical significance.  We have revised the Methods section accordingly:  “ *All variables with P-values less than 0.05 in the univariable complex samples logistic regression analysis, based on the Wald F test were included in the subsequent multivariable logistic regression model to determine factors associated with the prevalence of NCDs. The Wald F test was used as the omnibus criterion to assess the significance of predictors in the final model.”* | PG 8 LN 192-195 |
| 11 | There is no statement regarding missing data, whether it was present, its extent, or how it was handled in the analysis. | Thank you very much for the comment. In our dataset, variables included in the analysis contained minimal missing data. The Complex Samples logistic regression procedure in SPSS automatically performs listwise deletion, whereby cases with missing values on any variables included in a given model are excluded from that analysis. This approach preserves the integrity of the survey design because the procedure continues to apply sampling weights, clustering, and stratification to the remaining valid observations. The following statement has been added to the paper. “*Missing data were handled using listwise deletion within the SPSS Complex Samples procedures, which automatically exclude cases with missing values on any variables included in each model while preserving survey weights, clustering, and stratification. The proportion of missing data across included variables was minimal, and therefore no imputation was performed.*” | PG9 LN 203-207 |
| 12 | Please clarify whether all analyses, including both crude and adjusted models, accounted for the survey weights. | Thank you for this important observation. We confirm that all analyses including the univariable (crude) and multivariable (adjusted) logistic regression models were conducted using the SPSS Complex Samples module, which automatically incorporates the survey design features specified in a Complex Samples Plan File (CSPLAN file). This includes application of sampling weights, stratification, and clustering, as well as variance estimation via Taylor series linearization. Consequently, all reported parameter estimates, confidence intervals, and p‑values are design‑adjusted. We have clarified this in the Methods section as:  *“All crude and adjusted odds ratios were adjusted for the complex sampling design (weights, strata, and primary sampling units) and are presented with their 95% confidence intervals.”* | PG 9 LN 196-198 |
| 13 | Consider presenting Table 1 stratified by NCD status (no NCD vs. at least one NCD), in addition to the overall population distribution, as this would better inform adjusted associations and improve interpretability. | Thank you for the suggestion. We agree that stratifying Table 1 by NCD status enhances the interpretability of the findings. We have revised Table 1 to present the distribution of participants’ characteristics separately for individuals without any NCD and those with at least one NCD, alongside their design‑adjusted p‑values. These analyses were conducted using the SPSS Complex Samples procedures, which apply survey weights, clustering, and stratification to all descriptive comparisons. This stratified presentation provides clearer context for the adjusted associations reported in the multivariable models. | PG 11-12 |
| 14 | Tables 2 and 3: It is unclear why the sample size (n) varies across variables if an adjusted model was used. What is the missingness across all outcomes and variables. | Thank you very much for the comment. We must acknowledge the overall sample size is 14453 for Table 2 and 3. In Table 3. A few Variables that had a sample size of 14454 or 14452 was due to errors while creating that table where we rounded off unnecessarily and this has been corrected. For those with an overall sample size of less than 14453 (3 behavioral risk factors), it is because of missing data. These were added to the analysis regardless of their missingness because of their clinical significance in the multivariable model. During analysis these were deleted by the complex samples package by list wise deletion on a case by case basis. The Complex Samples logistic regression procedure applies **listwise deletion**, meaning that participants with missing values on any variable included in a model are excluded from that analysis while still accounting for survey weights, clustering, and stratification. Consequently, crude (bivariable) models, which were run separately for each predictor, had varying denominators depending on the amount of missing data for each variable. SPSS Complex Samples drops **cases with missing values on ANY variable in the final multivariable model**, which explains why “n” differs in some tables. This has been acknowledged in the methods section. | PG 11-14 |
| 15 | Line 306: “Napel” should be corrected to “Nepal.” | Thank you very much for the suggestion. We have corrected the spelling. | PG 29 LN 353 |
| 16 | Line 312: The study population includes only men aged 15–54 years. Given the strong age gradient of NCDs including hypertension and cancer, direct comparison with estimates that include older adults may not be appropriate and may partly explain the lower prevalence observed in this study. This should be acknowledged when interpreting difference in findings or risk patterns. | Thank you for this insightful comment. We agree that direct comparison of our NCD prevalence estimates with global figures requires caution, given that our study population consisted exclusively of men aged 15–54 years. NCDs such as hypertension and cancer exhibit a pronounced age gradient, with substantially higher prevalence among older adults. Therefore, the lower prevalence of hypertension and cancer observed in our study is likely influenced by the younger age structure of the sample. We have now revised the Discussion to explicitly acknowledge this issue and clarify that differences in age distribution partly explain the lower prevalence observed in our findings. We have added the following statement.  *“However, these comparisons should be interpreted cautiously because our study population included only men aged 15–54 years. Hypertension, as well as cancer, exhibits a strong age gradient, with prevalence rising sharply in older adults. Therefore, the lower prevalence observed in our sample likely reflects the younger age structure of the study population rather than true differences in underlying disease burden.”* | PG 29 LN 364-369 |
| 17 | In addition, when contexualizing findings in literature, it may be preferable to prioritize studies conducted in Kenya, East Africa or in countries with similar sociodemographic and epidemiologic profiles. Differences in age structure, cultural practices, health system and economic context may otherwise account for some of the observed variation. | Thank you for this valuable suggestion. We agree that contextualizing our results using studies conducted in populations with similar sociodemographic and epidemiological characteristics provides more meaningful interpretation. Direct comparison with global estimates may therefore not be appropriate.  Where appropriate we have revised the Discussion to prioritize evidence from Kenya and the broader East African region. Where we have compared with global prevalence estimates it is because of unavailable comparable literature in east Africa. | PG 28-36 |
| 18 | Many of the reported findings do not appear novel or unexpected. The discussion would benefit from a clearer statement of the study’s specific contribution to the existing literature. | Thank you for this insightful comment. We agree that the discussion should more clearly articulate the specific contribution of our study. While many of the associations observed are consistent with established patterns in the NCD literature, our study provides added value by focusing exclusively on men aged 15–54 years using a nationally representative, survey‑weighted dataset. This demographic is rarely examined separately in NCD research, despite the importance of early prevention among younger and middle‑aged men. Our analysis therefore helps to fill a gap in existing evidence by characterizing the burden and predictors of NCDs within this understudied population. We have revised the Discussion to highlight this contribution and to clarify how our findings complement and extend existing literature. We have added the following to the discussion;  ““*Although many of the identified risk factors for NCDs have been reported in prior studies, this analysis contributes to the literature by focusing specifically on men aged 15–54 years using a nationally representative data. Young and middle‑aged men remain an understudied population in NCD epidemiology, despite representing a key demographic for early prevention. Our findings therefore provide critical insights into the early distribution of NCDs and their associated risk factors in this age group, which can inform age‑ and* *gender‑responsive prevention strategies. Additionally, the use of Complex Samples methods allows for more accurate population‑level estimates within this demographic, filling an important evidence gap in the region.””* | PG 28-29 LN 342-350 |
|  | **REVIEWER # 3** |  |  |
| 1 | This manuscript addresses an important and timely public health issue—the burden and determinants of non-communicable diseases (NCDs) among men in Kenya using nationally representative 2022 KDHS data. The focus on men is particularly valuable, as male-specific epidemiological analyses remain limited in Sub-Saharan Africa. The topic is relevant, policy-sensitive, and potentially impactful. However, while the study demonstrates strong public health significance and uses a robust dataset, substantial revisions are required before the manuscript can meet the analytical rigor, conceptual depth, and narrative coherence expected in plos one journal. | Thank you very much for your compliment |  |
| 2 | The manuscript lacks a clear theoretical framework (e.g., Social Determinants of Health or Ecological Model). Integrating a conceptual model would improve coherence and strengthen scholarly contribution. | Thank you for this constructive suggestion. We agree that incorporating a theoretical framework would strengthen the coherence and scholarly contribution of the manuscript. We have integrated the Social Determinants of Health Model (SDOH) into the Methods to clarify how the variables included in the study reflect different levels of influence on NCD risk.  Specifically, Individual biological factors were listed as age, ethnicity). This was followed by Individual lifestyle factors which included tobacco use, past smoking, alcohol use, physical inactivity, and exercise). Social and community networks comprised marital status, religion, and access to media channels such as Television radio, internet, and newspaper. Lastly, living and working conditions included education, working status, wealth index, household size, region, residence.  Although the dataset did not include direct measures of socioeconomic, cultural and environmental conditions, we have discussed these conceptually to contextualize our findings. Incorporating the SDOH enhances the theoretical grounding of the study and clarifies the pathways through which the measured factors may contribute to NCD risk among men in Kenya. The following statement and diagram (Figure 1), has been added to the methods section of the manuscript:  “*The selection and organization of variables in this study were guided by Social Determinants of Health Model (SDoH) by Dahlgren & Whitehead 1991 ( Fig 1). Using the SDoH model we conceptualized the NCD risk as rising from a combination of Individual biological factors that are fixed (e.g., age, ethnicity), followed by other modifiable factors such as the individual lifestyle factors that can promote or damage health (e.g., tobacco use, past smoking, alcohol use, physical inactivity, and exercise), and the social and community networks that may or not provide the NCD related support (e.g., marital status, religion, and access to media channels such as Television radio, internet, and newspaper). Additionally, structural and environmental factors also termed as living and working conditions which shape daily life, resource access, and exposure to risks were included in the analysis and these included education, working status, wealth index, household size, region, residence. However, we did not include in our analysis socioeconomic, cultural and environmental conditions as these were absent in the dataset.* | PG 6-8 LN 149-182 |
| 3 | Greater clarity is needed regarding whether conditions were self-reported or physician-diagnosed. Potential underdiagnosis and reporting bias must be explicitly discussed. | Thank you for the comment. The NCDs analysed in the DHS dataset were **self-reported physician diagnoses**, meaning that participants reported whether a doctor or other qualified health professional had ever told them they had the listed condition. We agree that there may be potential **underdiagnosis** and **reporting bias**, which may lead to underestimation of true NCD prevalence. These biases are particularly relevant in LMIC settings where access to healthcare, diagnostic services, and routine screening are often limited. We have therefore added a paragraph in the limitations section explicitly discussing the likelihood of underdiagnosis and the inherent limitations associated with self-reported data, including recall and social desirability bias. The following statement has been added to the limitation section of this paper.  “*The fact that the study is based on retrospective information provided by the survey respondents, may have introduced recall, reporting (misclassification), and socio-desirability bias since it did not allow verification of clinical assessments or disease severity. Thus, our study outcomes are subject to under-diagnosis*.” | PG 36 LN 535-538 |
| 4 | Provide more details on sampling weights, model diagnostics, and handling of confounders | Thank you for the comments. We clarified that the DHS individual men’s sample weight (mv005) was normalized and applied in all analyses. In SPSS Complex Samples, the weight variable, primary sampling units (mv021), and stratification variable (mv022) were explicitly specified to account for the DHS’s two‑stage stratified cluster sampling design. We have added explanations of the purpose of the weights (to correct for unequal probability of selection and nonresponse).  We also added a description of diagnostic procedures performed. These include checking for multicollinearity using Variance Inflation Factors (VIF), examination of model fit indices, and assessment of residuals.  lastly, we clarified that confounders were selected a priori based on existing literature and conceptual frameworks on NCD determinants. All potential confounders were retained in multivariable models regardless of statistical significance to minimize omitted variable bias. | PG 8-9 LN 184-207 |
| 5 | Consider deeper health systems implications and clarify the definition of multiple NCDs. | Thank you very much for the comment or suggestions. We have reflected on the deeper health systems implications for each results and included them in the discussion and conclusion sections of this paper. We have defined multiple NCDs in the methods section and discussed it implications in global health | PG 6 LN 145-147, Pg 28-38 |
| 6 | The higher burden in rural areas deserves deeper exploration. | Thank you for the comment. We agree that the higher NCD risk observed in less urbanized regions warrants further explanation. Evidence shows that NCD risk in Africa is not exclusively an urban phenomenon; rural populations may have significant exposure to risk factors such as tobacco and alcohol use, limited access to preventive services, and delayed diagnosis. Studies analyzing NCD risk‑factor patterns in Africa indicate that rural dwellers may have equal or, in some cases, higher levels of certain NCD risks compared to urban populations. We have expanded the discussion section to incorporate these contextual factors and to provide a more nuanced interpretation of our findings.  The following statement has been added to the paper  “*Although NCDs are often associated with urbanization, our findings showing elevated NCD risk among men in less‑urbanized regions such as Nyanza, North Eastern, and Rift Valley are consistent with emerging evidence that rural populations in sub‑Saharan Africa also experience substantial NCD risk. Studies demonstrate that rural residents may exhibit significant exposure to risk factors including tobacco and alcohol use and face structural barriers such as limited access to screening and essential NCD services, contributing to delayed diagnosis and poorer outcomes*” | PG 32 LN 421-430 |
| 7 | Reduce repetition in the discussion section. | Thank you very much for the comment or suggestions. Where necessary, we have edited and reduced the repetition in the discussion section by further summarizing them | PG 28-36 |
| 8 | Expand limitations to include potential residual confounding. | Thank you for this important comment. We agree that residual confounding is a potential limitation of our study. Although we adjusted for a wide range of structural, individual, behavioral, and community factors available in the KDHS dataset, we acknowledge that the survey does not capture several established NCD risk factors such as dietary patterns, body weight salt intake, cholesterol levels, occupational exposures, air pollution, family history, or detailed measures of stress. In addition, some variables (e.g., physical activity, sedentary time, perceived health) are self‑reported and may be subject to misclassification. Therefore, unmeasured or imperfectly measured confounders may still influence the observed associations. We have now expanded the Limitations section to reflect this.  “*Although our multivariable models adjusted for multiple structural, individual, behavioral, and community factors, the possibility of residual confounding cannot be excluded. The KDHS did not collect several important NCD risk determinants, including dietary composition, salt intake, cholesterol levels, genetic predisposition, environmental exposures (e.g., air pollution), detailed occupational risks, and stress levels. Additionally, some covariates such as physical activity, sedentary behavior, and perceived health are self‑reported and may be prone to misclassification. As a result, unmeasured or imperfectly measured confounders may influence the associations observed in this study.”* | PG 37 LN 543-550 |
| 9 | The use of the KDHS data is one of the major strengths in this study and it has strong potential but requires substantial revision | Thank you very much for the compliment. The KDHS data strength has been acknowledged in the paper and substantial revision to the paper has been made as recommended by the reviewers. |  |
