# Supplementary material for: Prevalence and associated factors of non-communicable diseases among men: A cross-sectional analysis of Kenya Demographic and Health Survey 2022 data
Source: PLoS One. 2026 Jul 20;21(7):e0327266. doi: 10.1371/journal.pone.0327266 (PMC13384318; doi:10.1371/journal.pone.0327266)
Supplement: Attachment — Submitted filename: PLOS_Review_AS.docx [file pone.0327266.s003.docx]

**Reviewers report**

The manuscript titled “Prevalence and associated factors of non-communicable diseases among men in Kenya: an analysis of the 2022 Kenya Demographic and Health Survey” is a well written article that explored the prevalence and social, demographic, behavioral factors of NCD in Kenya. The study was done on a larger sample size, using appropriate statistical methods making it relevant for public health intervention to tackle NCD. It also satisfies the PLOS ONE criteria for publication. However, there are some minor issues that need to be corrected and clarified.

1. **Title**

- The study was conducted among Kenyan Male population. Please justify why the study excluded women and the relevance of this segmented study for the policy.

2. **Abstract**

- Page 2: line # 49-50: This study has exclusively assessed the prevalence and associated factors of NCD among Men. And the authors concluded that *“The overall prevalence of NCDs among men is relatively lower than that of Kenyan women.”* How this conclusion is possible without studying the female population? The conclusion part of an article should be based on the findings of its own result.

3. **Result**

- Page 9, Table 1: The table title doesn’t fully explain the content. It says “socio-demographic characteristics …” But it has also behavioral and clinical variables. Consider revision.
- Page 9, Table 1: The table is too long to the reader. Consider splitting.
- Page 11, Table 2: The total number of participants **living with ≥2 NCDs** is reported as *n = 14174 (1.9%).* However, if 14174 of them have ≥2 NCDs, it will be it will be 98.1%. Please revise the number.
- Page 12, line # 225: Add space between aOR and the number.
- Page 14, Line # 277: The study found that religion was among one of the statistically significant variables. Since religion can be sensitive issue in many contexts, please proof that it will not be point of dispute
