# Supplementary material for: Prevalence and associated factors of non-communicable diseases among men: A cross-sectional analysis of Kenya Demographic and Health Survey 2022 data
Source: PLoS One. 2026 Jul 20;21(7):e0327266. doi: 10.1371/journal.pone.0327266 (PMC13384318; doi:10.1371/journal.pone.0327266)
Supplement: Attachment — Submitted filename: Response to Reviewers..docx [file pone.0327266.s006.docx]

| # | Comment | Response | Page/Line Number |
| --- | --- | --- | --- |
| 1 | Provide evidence of your variance inflation factor (VIF) | A citation has been added to the paper. We have added the following citation “*Johnston, R., Jones, K., & Manley, D. (2018). Confounding and collinearity in regression analysis: a cautionary tale and an alternative procedure, illustrated by studies of British voting behaviour. Quality & quantity, 52(4), 1957-1976*.” | Page 9, LN 200, Page 40, citation 20 |
| 2 | Concerning Data availability, you stated that 'Yes - all data are fully available without restriction,' where exactly can the data be accessed? | The following statement in the manuscript has been added to the online submission system. However, the statement shows up below the ethics statement.  “*Third-party data was obtained for this study from The DHS Program (https://dhsprogram.com/). Data may be requested from the DHS Program after creating an account and submitting a concept note. More access information can be found on the DHS Program website (https://dhsprogram.com/data/Access-Instructions.cfm). The data set is openly available upon permission from the MEASURE DHS website (https://www.dhsprogram.com/data/available-datasets.cfm). The authors confirm that interested researchers would be able to access these data in the same manner as the authors. The authors also confirm that they had no special access privileges that others would not have*.” | Pg 38 LN 584-589 |
| 3 | Some words are in British as well as US spelling. Examples include analyse/analyze, Behavioural/behavioral etc. Please adopt one. | All words have been revised through out the paper to reflect the American English language | LN 35, 102, 138, 174, 176, 232, Table 1,3,4 |

1
