# Supplementary material for: Prevalence and associated factors of non-communicable diseases among men: A cross-sectional analysis of Kenya Demographic and Health Survey 2022 data
Source: PLoS One. 2026 Jul 20;21(7):e0327266. doi: 10.1371/journal.pone.0327266 (PMC13384318; doi:10.1371/journal.pone.0327266)
Supplement: Attachment — Submitted filename: Response_to_Reviewers._auresp_3.docx [file pone.0327266.s007.docx]

| # | Comment | Response | Page/Line Number |
| --- | --- | --- | --- |
| 1 | Regarding the evidence of your variance inflation factor (VIF), I suggest you provide a Table that shows the results of the VIFs for the various variables, as well as the mean values. Provide this as a supplementary file | Thanks, editor, for this comment. A new Supplementary Table (S1 Table) has been added reporting VIFs and tolerance values for all predictors, along with the mean VIF. All VIF values were below commonly accepted thresholds (<5), indicating no problematic multicollinearity. | Page 9 LN 201 and pg 43 |
| 2 | Authors are also advised to use STROBE guidelines for reporting of observational studies. | A new Supplementary Table (S1 STROBE Checklist) has been added, indicating where all the recommendations of STROBE have been incorporated in the manuscript. | Pg 9 LN 209-210 and pg 43 |

1
