# Supplementary material for: Prevalence and associated factors of non-communicable diseases among men: A cross-sectional analysis of Kenya Demographic and Health Survey 2022 data
Source: PLoS One. 2026 Jul 20;21(7):e0327266. doi: 10.1371/journal.pone.0327266 (PMC13384318; doi:10.1371/journal.pone.0327266)
Supplement: Attachment — Submitted filename: Response_to_Reviewers._auresp_4.docx [file pone.0327266.s008.docx]

| # | Comment | Response | Page/Line Number |
| --- | --- | --- | --- |
| 1 | “ Pg 1-2, LN 3, 35” The study design is not indicated in the title nor the abstract | Thanks for the comment. The term cross-sectional survey has been added to the title, abstract, and methods. | Pg 1, LN 2  Pg 2, LN 34-35  Pg 5, LN114-115 |
| 2 | Indicate the total number of respondents in Table 1 | Thanks for the comment. The total number of respondents has been added to table 1. | Table 1, Pg 11 |
| 3 | The frequencies for the following variables: exercise minutes per week, past smoking variable, number of hours per week seated, health status, do not sum up to the total number of respondent (N=14453). | Thanks for the comment once again. For the variable “health status”, the mistake was due to the typo error while creating that table where we rounded off unnecessarily and this has been corrected which has been corrected. Past smoking has been eliminated based on new multicollinearity diagnostic statistics. The other variables (exercise minutes per week, number of hours per week seated), it was because of the missing figures. These were added to the analysis regardless of their missingness because of their clinical significance in the multivariable model. During analysis these were deleted by the complex samples package by list wise deletion on a case-by-case basis. The following disclaimer has been added below table 1. “*The variable does not add up to the total sample size of 14453 and were added to the analysis regardless of their missing data because of their clinical significance in the multivariable model”* | Pg 12, LN244-246, Table 1 |
| 4 | Please, the justification for dropping the smoking tobacco variable in the VIF is not valid. I presume the variable was dropped because of an issue of multicollinearity. Meanwhile it was included in both regression analysis. Using both past smoking and tobacco use is problematic. Is the past smoking variable a sub-set of tobacco use? It is likely past users are included in category of those who answered no to tobacco use. Why is there a drastic reduction in the frequency (if it’s not typo? Include both variables in the VIF for verification. I suggest you either drop past smoking or recode the two variables (past smoking and tobacco use) into one variable (never smoked, former smoker, current smoker). | Thanks for the comments. Tobacco use was a composite variable computed from two variables namely currently smoking daily cigarettes/other tobacco products AND using daily smokeless tobacco (current user). Whereas past smoking tobacco included respondents who smoked daily in the past but are now currently non-smokers (former users). It is likely that past users were included in category of those who answered no to tobacco use. Therefore, we have decided to drop the past smoking variable and repeated the multivariable analysis where it was included (lung diseases). We have also updated the multicollinearity diagnostic supplementary table 1. All necessary changes in line with this comment in the methods and results section have been made. | Pg 7, LN174-175,  Pg 10, 233,  Pg 14-15, LN 274-275, 283, 293, 300, 328-329.  Pg 31-34, LN 422, 441, 491-492, 527-528  Table 1, 2, 3. |
| 5 | Rename the supplementary files as S1 and S2 | Thanks for the comment. The supplementary files have been renamed as advised. | Pg 9, 201 and 209, Pg 43 |
| 6 | Recheck the line numbers reported in the Strobe checklist for accuracy. | Thanks for the comment. We have double checked the checklist to ensure that the page/line number correspond to the content of the STROBE checklist | S2 Table |

1
