# Supplementary material for: Prevalence and associated factors of non-communicable diseases among men: A cross-sectional analysis of Kenya Demographic and Health Survey 2022 data
Source: PLoS One. 2026 Jul 20;21(7):e0327266. doi: 10.1371/journal.pone.0327266 (PMC13384318; doi:10.1371/journal.pone.0327266)
Supplement: Attachment — Submitted filename: Response_to_Reviewers._auresp_5.docx [file pone.0327266.s009.docx]

| # | Comment | Response | Page/line |
| --- | --- | --- | --- |
| 1 | Thank you for your response to the earlier comments. However, there are further statistical issues that need to be addressed.  How do you conclude or interpret your study if there were missing data and you included the variables without addressing those missing cases? How did you treat or address your missing cases? If you addressed them well, the sample size would have been equal , particularly at the bivariate level and regression levels. Please address the issue of missing cases in your data and redo the analysis. Please provide a flow chart of how you arrived at your new sample size afterwards. | Thank you very much for the comment.  There were two behavioral variables with missing data namely exercise and sedentary lifestyle. Since sedentary lifestyle can be a proxy for engaging in exercise, we have decided to retain the variable. Exercise minutes per week has been eliminated due to a large missing data (>15%). We have also deleted the 14 participants with missing figures on sedentary lifestyle and reduced the sample size to14,439. The analysis has been re-run based on the newer variables and sample size.  We have made all necessary changes throughout the manuscript  We have also re-run the multicollinearity diagnostics  The statement on missing data has been edited as “*Lastly, variables with substantial missing data (≥15%) such as the number of minutes exercised per week were excluded in the analysis regardless of their clinical significance. Variables with minimal missing were handled on a case-by-case basis using list*-*wise deletion of participants with missing data (n=14). This brought the final sample size to 14, 439 from 14,453 participants who were interviewed.*”  We believe given the above explanation a flow chart may not be necessary. | Pg 5, LN 127-130  Page 6, LN 153  Pg 7 LN 173-174  Page 9, LN 202-207  Pg 10-28  Table 1,2, 3, 4, Figure 1 |
| 2 | Also, Table two does not sum up to 14453 as indicated in the table (However, this sample will change after addressing the missing cases). I expected to see a category of those who had no condition, or is your study amongst those who had a condition? | Thank you very much for the comment.  The previous variables provided under table 2, were for those who had the condition with the expectation that if one used the overall sample size, he/she could arrive at the ‘no condition’ for each variable.  We have however added that column for the ‘no condition’ | Table 2 |
| 3 | Importantly, respondents with two or more conditions sum up to 246 and not 275 as indicated (This is likely to change after addressing the missing cases | Thank you very much for the comment.  The Figure for multiple NCDs has been updated to 279. If computed out of 14,439, it gives a prevalence of 1.9%. | Table 2 |
| 4 | The sample sizes are not equal in all stages of the analysis. Please indicate the sample size at each level of the analysis for readers to understand the variations in the methodology section. | Thank you very much for the comment. The sample size has been made equal after eliminating missing figures. All analysis has is now based on 14,439. | Table 1, 2, 3, 4 |
