# Supplementary material for: Prevalence and associated factors of non-communicable diseases among men: A cross-sectional analysis of Kenya Demographic and Health Survey 2022 data
Source: PLoS One. 2026 Jul 20;21(7):e0327266. doi: 10.1371/journal.pone.0327266 (PMC13384318; doi:10.1371/journal.pone.0327266)
Supplement: Peer review history

# Peer review: Decision Letter 0

**DOI:** 10.1371/journal.pone.0327266.r001

**Authors:** Grace Afrifa-Anane

9 Mar 2026

PONE-D-25-31448

Prevalence and associated factors of non-communicable diseases among men in Kenya: an analysis of the 2022 Kenya Demographic and Health Survey

PLOS One

Dear Dr. Asiimwe,

Thank you for submitting your manuscript to PLOS ONE. After careful consideration, we feel that it has merit but does not fully meet PLOS ONE’s publication criteria as it currently stands. Therefore, we invite you to submit a revised version of the manuscript that addresses the points raised during the review process.

-->Please address the following comments:

1. Tighten up the research gap by highlighting the sex-specific rationale

2. Table 3 is compacted, which makes it quite difficult to comprehend. I suggest you  revise it by focusing on the adjusted odds ratio and associated confidence intervals

2. Revise your conclusion based on findings from your study to avoid establishing a causal relationship

Please submit your revised manuscript by Apr 23 2026 11:59PM. If you will need more time than this to complete your revisions, please reply to this message or contact the journal office at plosone@plos.org. When you're ready to submit your revision, log on to https://www.editorialmanager.com/pone/ and select the 'Submissions Needing Revision' folder to locate your manuscript file.

Please include the following items when submitting your revised manuscript:

- A letter that responds to each point raised by the academic editor and reviewer(s). You should upload this letter as a separate file labeled 'Response to Reviewers'.
- A marked-up copy of your manuscript that highlights changes made to the original version. You should upload this as a separate file labeled 'Revised Manuscript with Track Changes'.
- An unmarked version of your revised paper without tracked changes. You should upload this as a separate file labeled 'Manuscript'.

-->If you would like to make changes to your financial disclosure, please include your updated statement in your cover letter. Guidelines for resubmitting your figure files are available below the reviewer comments at the end of this letter.

If applicable, we recommend that you deposit your laboratory protocols in protocols.io to enhance the reproducibility of your results. Protocols.io assigns your protocol its own identifier (DOI) so that it can be cited independently in the future. For instructions see: https://journals.plos.org/plosone/s/submission-guidelines#loc-laboratory-protocols. Additionally, PLOS ONE offers an option for publishing peer-reviewed Lab Protocol articles, which describe protocols hosted on protocols.io. Read more information on sharing protocols at https://plos.org/protocols?utm_medium=editorial-email&utm_source=authorletters&utm_campaign=protocols.

We look forward to receiving your revised manuscript.

Kind regards,

Grace Frempong Afrifa-Anane, Ph.D

Guest Editor

PLOS One

Journal Requirements:

When submitting your revision, we need you to address these additional requirements.

1. Please ensure that your manuscript meets PLOS ONE's style requirements, including those for file naming. The PLOS ONE style templates can be found at

https://journals.plos.org/plosone/s/file?id=wjVg/PLOSOne_formatting_sample_main_body.pdf and

https://journals.plos.org/plosone/s/file?id=ba62/PLOSOne_formatting_sample_title_authors_affiliations.pdf

2. Please note that your Data Availability Statement is currently missing the DOI/accession number of each dataset or a direct link to access each database. If your manuscript is accepted for publication, you will be asked to provide these details on a very short timeline. We therefore suggest that you provide this information now, though we will not hold up the peer review process if you are unable.

3. If the reviewer comments include a recommendation to cite specific previously published works, please review and evaluate these publications to determine whether they are relevant and should be cited. There is no requirement to cite these works unless the editor has indicated otherwise.

4. Please review your reference list to ensure that it is complete and correct. If you have cited papers that have been retracted, please include the rationale for doing so in the manuscript text, or remove these references and replace them with relevant current references. Any changes to the reference list should be mentioned in the rebuttal letter that accompanies your revised manuscript. If you need to cite a retracted article, indicate the article’s retracted status in the References list and also include a citation and full reference for the retraction notice.

[Note: HTML markup is below. Please do not edit.]

Reviewers' comments:

Reviewer's Responses to Questions

**Comments to the Author**

1. Is the manuscript technically sound, and do the data support the conclusions?

The manuscript must describe a technically sound piece of scientific research with data that supports the conclusions. Experiments must have been conducted rigorously, with appropriate controls, replication, and sample sizes. The conclusions must be drawn appropriately based on the data presented.

Reviewer #1: Yes

Reviewer #2: Partly

Reviewer #3: Yes

**********

2. Has the statistical analysis been performed appropriately and rigorously?

Reviewer #1: Yes

Reviewer #2: No

Reviewer #3: Yes

**********

3. Have the authors made all data underlying the findings in their manuscript fully available?

The PLOS Data policy requires authors to make all data underlying the findings described in their manuscript fully available without restriction, with rare exception (please refer to the Data Availability Statement in the manuscript PDF file). The data should be provided as part of the manuscript or its supporting information, or deposited to a public repository. For example, in addition to summary statistics, the data points behind means, medians and variance measures should be available. If there are restrictions on publicly sharing data—e.g. participant privacy or use of data from a third party—those must be specified.

Reviewer #1: Yes

Reviewer #2: Yes

Reviewer #3: Yes

**********

4. Is the manuscript presented in an intelligible fashion and written in standard English?

PLOS ONE does not copyedit accepted manuscripts, so the language in submitted articles must be clear, correct, and unambiguous. Any typographical or grammatical errors should be corrected at revision, so please note any specific errors here.

Reviewer #1: Yes

Reviewer #2: Yes

Reviewer #3: Yes

**********

5. Review Comments to the Author

Please use the space provided to explain your answers to the questions above. You may also include additional comments for the author, including concerns about dual publication, research ethics, or publication ethics. (Please upload your review as an attachment if it exceeds 20,000 characters)

Reviewer #1: Reviewers report

The manuscript titled “Prevalence and associated factors of non-communicable diseases among men in Kenya: an analysis of the 2022 Kenya Demographic and Health Survey” is a well written article that explored the prevalence and social, demographic, behavioral factors of NCD in Kenya. The study was conducted on a larger sample size, using appropriate statistical methods making it relevant for public health interventions to tackle NCD. It also satisfies the PLOS ONE criteria for publication. However, there are some minor issues that need to be corrected and clarified.

1. Title

• The study was conducted among the Kenyan Male population. Please justify why the study excluded women and the relevance of this segmented study for the policy.

2. Abstract

• Page 2: line # 49-50: This study has exclusively assessed the prevalence and associated factors of NCD among Men. And the authors concluded that “The overall prevalence of NCDs among men is relatively lower than that of Kenyan women.” How this conclusion is possible without studying the female population? The conclusion part of an article should be based on the findings of its own result.

3. Result

• Page 9, Table 1: The table title doesn’t fully explain the content. It says “socio-demographic characteristics …” But it has also behavioral and clinical variables. Consider revision.

• Page 9, Table 1: The table is too long to the reader. Consider splitting.

• Page 11, Table 2: The total number of participants living with ≥2 NCDs is reported as n = 14174 (1.9%). However, if 14174 of them have ≥2 NCDs, it will be it will be 98.1%. Please revise the number.

• Page 12, line # 225: Add space between aOR and the number.

• Page 14, Line # 277: The study found that religion was among one of the statistically significant variables. Since religion can be sensitive issue in many contexts, please proof that it will not be point of dispute

Reviewer #2: Line 51: Given the cross-sectional design, recommendations should be framed more cautiously, avoiding causal language (e.g., implying that doing X will reduce Y). This also applies to the discussion and conclusion.

Line 49: The current study does not support this conclusion.

Line 54: Although the findings indicate higher NCD prevalence among wealthier and more educated men, recommendations for healthy lifestyle promotion should apply broadly across socioeconomic groups. The recommendations should be framed more inclusively.

Line 56: While the conclusions may be reasonable in a broader public health context, they extend beyond the findings of this study. The analysis does not examine media content, health messaging, or stigma.

Line 60: The introduction outlines the burden of NCDs, differences in life expectancy by sex, and patterns of risk behaviors. However, it does not clearly explain why the study focuses specifically on men. Clarifying the sex-specific rationale would strengthen the argument and make the study objective more convincing.

Line 115: Please clarify how the reported 98% response rate was calculated. According to the 2022 KDHS report, 16,552 men aged 15–54 were eligible for individual interviews, and 14,453 were successfully interviewed (response rate: 87%). Additionally, please specify the inclusion and exclusion criteria applied in the analysis.

Line 124: The included NCDs are heterogeneous. It would be helpful to provide a clearer rationale to justify their inclusion, particularly given the 15–54 year age group.

Line 164: “multivariate” should be replaced with “multivariable,” considering separate regression models were fitted for each NCD outcome rather than modeling multiple outcomes simultaneously.

Line 166: The term “simple multivariate logistic regression” is not standard.

Line 166: Were predictors retained based on an omnibus test (e.g., likelihood ratio or Wald test)

There is no statement regarding missing data, whether it was present, its extent, or how it was handled in the analysis.

Please clarify whether all analyses, including both crude and adjusted models, accounted for the survey weights.

Consider presenting Table 1 stratified by NCD status (no NCD vs. at least one NCD), in addition to the overall population distribution, as this would better inform adjusted associations and improve interpretability.

Tables 2 and 3: It is unclear why the sample size (n) varies across variables if an adjusted model was used. What is the missingness across all outcomes and variables.

Line 306: “Napel” should be corrected to “Nepal.”

Line 312: The study population includes only men aged 15–54 years. Given the strong age gradient of NCDs including hypertension and cancer, direct comparison with estimates that include older adults may not be appropriate and may partly explain the lower prevalence observed in this study. This should be acknowledged when interpreting difference in findings or risk patterns.

In addition, when contexualizing findings in literature, it may be preferable to prioritize studies conducted in Kenya, East Africa or in countries with similar sociodemographic and epidemiologic profiles. Differences in age structure, cultural practices, health system and economic context may otherwise account for some of the observed variation.

Many of the reported findings do not appear novel or unexpected. The discussion would benefit from a clearer statement of the study’s specific contribution to the existing literature.

Reviewer #3: This manuscript addresses an important and timely public health issue—the burden and determinants of non-communicable diseases (NCDs) among men in Kenya using nationally representative 2022 KDHS data. The focus on men is particularly valuable, as male-specific epidemiological analyses remain limited in Sub-Saharan Africa. The topic is relevant, policy-sensitive, and potentially impactful.

However, while the study demonstrates strong public health significance and uses a robust dataset, substantial revisions are required before the manuscript can meet the analytical rigor, conceptual depth, and narrative coherence expected in plos one journal.

Major comments

1-The manuscript lacks a clear theoretical framework (e.g., Social Determinants of Health or Ecological Model). Integrating a conceptual model would improve coherence and strengthen scholarly contribution.

2-Greater clarity is needed regarding whether conditions were self-reported or physician-diagnosed. Potential underdiagnosis and reporting bias must be explicitly discussed.

3-Provide more details on sampling weights, model diagnostics, and handling of confounders.

4-Consider deeper health systems implications and clarify the definition of multiple NCDs.

5-The higher burden in rural areas deserves deeper exploration.

6-Reduce repetition in the discussion section.

7-Expand limitations to include potential residual confounding.

The use of the KDHS data is one of the major strengths in this study and it has strong potential but requires substantial revision.

**********

6. PLOS authors have the option to publish the peer review history of their article (what does this mean?). If published, this will include your full peer review and any attached files.

If you choose “no”, your identity will remain anonymous but your review may still be made public.

**Do you want your identity to be public for this peer review?**  For information about this choice, including consent withdrawal, please see our Privacy Policy.

Reviewer #1: **Yes:**  Abel Shita

Reviewer #2: No

Reviewer #3: No

**********

[NOTE: If reviewer comments were submitted as an attachment file, they will be attached to this email and accessible via the submission site. Please log into your account, locate the manuscript record, and check for the action link "View Attachments". If this link does not appear, there are no attachment files.]

To ensure your figures meet our technical requirements, please review our figure guidelines: https://journals.plos.org/plosone/s/figures

You may also use PLOS’s free figure tool, NAAS, to help you prepare publication quality figures: https://journals.plos.org/plosone/s/figures#loc-tools-for-figure-preparation.

NAAS will assess whether your figures meet our technical requirements by comparing each figure against our figure specifications.

Attachment Submitted filename: PLOS_Review_AS.docx

# Author comment: Author response to Decision Letter 1

**DOI:** 10.1371/journal.pone.0327266.r002

31 Mar 2026

Kindly see the attached document entitled " response to reviewer comments" below

Attachment Submitted filename: Response to Reviewers.docx

# Peer review: Decision Letter 1

**DOI:** 10.1371/journal.pone.0327266.r003

**Authors:** Grace Afrifa-Anane, Grace Afrifa-Anane

27 Apr 2026

PONE-D-25-31448R1

Prevalence and associated factors of non-communicable diseases among men in Kenya: an analysis of the 2022 Kenya Demographic and Health Survey

PLOS One

Dear Dr. Asiimwe,

Thank you for submitting your manuscript to PLOS ONE. After careful consideration, we feel that it has merit but does not fully meet PLOS ONE’s publication criteria as it currently stands. Therefore, we invite you to submit a revised version of the manuscript that addresses the points raised during the review process.

Please submit your revised manuscript by Jun 11 2026 11:59PM. If you will need more time than this to complete your revisions, please reply to this message or contact the journal office at plosone@plos.org. When you're ready to submit your revision, log on to https://www.editorialmanager.com/pone/ and select the 'Submissions Needing Revision' folder to locate your manuscript file.

Please include the following items when submitting your revised manuscript:

- A letter that responds to each point raised by the academic editor and reviewer(s). You should upload this letter as a separate file labeled 'Response to Reviewers'.
- A marked-up copy of your manuscript that highlights changes made to the original version. You should upload this as a separate file labeled 'Revised Manuscript with Track Changes'.
- An unmarked version of your revised paper without tracked changes. You should upload this as a separate file labeled 'Manuscript'.

If you would like to make changes to your financial disclosure, please include your updated statement in your cover letter. Guidelines for resubmitting your figure files are available below the reviewer comments at the end of this letter.

If applicable, we recommend that you deposit your laboratory protocols in protocols.io to enhance the reproducibility of your results. Protocols.io assigns your protocol its own identifier (DOI) so that it can be cited independently in the future. For instructions see: https://journals.plos.org/plosone/s/submission-guidelines#loc-laboratory-protocols. Additionally, PLOS ONE offers an option for publishing peer-reviewed Lab Protocol articles, which describe protocols hosted on protocols.io. Read more information on sharing protocols at https://plos.org/protocols?utm_medium=editorial-email&utm_source=authorletters&utm_campaign=protocols.

As the corresponding author, your ORCID iD is verified in the submission system and will appear in the published article. PLOS supports the use of ORCID, and we encourage all coauthors to register for an ORCID iD and use it as well. Please encourage your coauthors to verify their ORCID iD within the submission system before final acceptance, as unverified ORCID iDs will not appear in the published article. *Only*  the individual author can complete the verification step; PLOS staff *cannot*  verify ORCID iDs on behalf of authors.

We look forward to receiving your revised manuscript.

Kind regards,

Grace Frempong Afrifa-Anane, Ph.D

Guest Editor

PLOS One

Journal Requirements:

1. If the reviewer comments include a recommendation to cite specific previously published works, please review and evaluate these publications to determine whether they are relevant and should be cited. There is no requirement to cite these works unless the editor has indicated otherwise.

2. Please review your reference list to ensure that it is complete and correct. If you have cited papers that have been retracted, please include the rationale for doing so in the manuscript text, or remove these references and replace them with relevant current references. Any changes to the reference list should be mentioned in the rebuttal letter that accompanies your revised manuscript. If you need to cite a retracted article, indicate the article’s retracted status in the References list and also include a citation and full reference for the retraction notice.

Additional Editor Comments:

1. Provide evidence of your variance inflation factor (VIF)

2. Concerning Data availability, you stated that 'Yes - all data are fully available without restriction,' where exactly can the data be accessed?

3. Some words are in British as well as US spelling. Examples include analyse/analyze, Behavioural/behavioral etc. Please adopt one.

Reviewers' comments:

Reviewer's Responses to Questions

**Comments to the Author**

1. If the authors have adequately addressed your comments raised in a previous round of review and you feel that this manuscript is now acceptable for publication, you may indicate that here to bypass the “Comments to the Author” section, enter your conflict of interest statement in the “Confidential to Editor” section, and submit your "Accept" recommendation.

Reviewer #1: All comments have been addressed

Reviewer #2: All comments have been addressed

Reviewer #3: All comments have been addressed

**********

2. Is the manuscript technically sound, and do the data support the conclusions?

The manuscript must describe a technically sound piece of scientific research with data that supports the conclusions. Experiments must have been conducted rigorously, with appropriate controls, replication, and sample sizes. The conclusions must be drawn appropriately based on the data presented.

Reviewer #1: Yes

Reviewer #2: Yes

Reviewer #3: Yes

**********

3. Has the statistical analysis been performed appropriately and rigorously?

Reviewer #1: Yes

Reviewer #2: Yes

Reviewer #3: Yes

**********

4. Have the authors made all data underlying the findings in their manuscript fully available?

The PLOS Data policy requires authors to make all data underlying the findings described in their manuscript fully available without restriction, with rare exception (please refer to the Data Availability Statement in the manuscript PDF file). The data should be provided as part of the manuscript or its supporting information, or deposited to a public repository. For example, in addition to summary statistics, the data points behind means, medians and variance measures should be available. If there are restrictions on publicly sharing data—e.g. participant privacy or use of data from a third party—those must be specified.

Reviewer #1: Yes

Reviewer #2: Yes

Reviewer #3: (No Response)

**********

5. Is the manuscript presented in an intelligible fashion and written in standard English?

PLOS ONE does not copyedit accepted manuscripts, so the language in submitted articles must be clear, correct, and unambiguous. Any typographical or grammatical errors should be corrected at revision, so please note any specific errors here.

Reviewer #1: Yes

Reviewer #2: Yes

Reviewer #3: Yes

**********

6. Review Comments to the Author

Please use the space provided to explain your answers to the questions above. You may also include additional comments for the author, including concerns about dual publication, research ethics, or publication ethics. (Please upload your review as an attachment if it exceeds 20,000 characters)

Reviewer #1: (No Response)

Reviewer #2: (No Response)

Reviewer #3: (No Response)

**********

7. PLOS authors have the option to publish the peer review history of their article (what does this mean?). If published, this will include your full peer review and any attached files.

If you choose “no”, your identity will remain anonymous but your review may still be made public.

**Do you want your identity to be public for this peer review?**  For information about this choice, including consent withdrawal, please see our Privacy Policy.

Reviewer #1: No

Reviewer #2: No

Reviewer #3: No

**********

[NOTE: If reviewer comments were submitted as an attachment file, they will be attached to this email and accessible via the submission site. Please log into your account, locate the manuscript record, and check for the action link "View Attachments". If this link does not appear, there are no attachment files.]

To ensure your figures meet our technical requirements, please review our figure guidelines: https://journals.plos.org/plosone/s/figures

You may also use PLOS’s free figure tool, NAAS, to help you prepare publication quality figures: https://journals.plos.org/plosone/s/figures#loc-tools-for-figure-preparation.

NAAS will assess whether your figures meet our technical requirements by comparing each figure against our figure specifications.

# Author comment: Author response to Decision Letter 2

**DOI:** 10.1371/journal.pone.0327266.r004

28 Apr 2026

We have attached a file labeled "response to reviewer comments"

Attachment Submitted filename: Response to Reviewers..docx

# Editor report: Decision Letter 2

**DOI:** 10.1371/journal.pone.0327266.r005

**Authors:** Grace Afrifa-Anane, Grace Afrifa-Anane, Grace Afrifa-Anane

30 Apr 2026

PONE-D-25-31448R2

Prevalence and associated factors of non-communicable diseases among men in Kenya: an analysis of the 2022 Kenya Demographic and Health Survey

PLOS One

Dear Dr. Asiimwe,

Thank you for submitting your manuscript to PLOS ONE. After careful consideration, we feel that it has merit but does not fully meet PLOS ONE’s publication criteria as it currently stands. Therefore, we invite you to submit a revised version of the manuscript that addresses the points raised during the review process.

Please submit your revised manuscript by Jun 13 2026 11:59PM. If you will need more time than this to complete your revisions, please reply to this message or contact the journal office at plosone@plos.org. When you're ready to submit your revision, log on to https://www.editorialmanager.com/pone/ and select the 'Submissions Needing Revision' folder to locate your manuscript file.

Please include the following items when submitting your revised manuscript:

- A letter that responds to each point raised by the academic editor and reviewer(s). You should upload this letter as a separate file labeled 'Response to Reviewers'.
- A marked-up copy of your manuscript that highlights changes made to the original version. You should upload this as a separate file labeled 'Revised Manuscript with Track Changes'.
- An unmarked version of your revised paper without tracked changes. You should upload this as a separate file labeled 'Manuscript'.
- STROBES guideline checklist as a supplementary file
- VIF results as a supplementary file

If you would like to make changes to your financial disclosure, please include your updated statement in your cover letter. Guidelines for resubmitting your figure files are available below the reviewer comments at the end of this letter.

If applicable, we recommend that you deposit your laboratory protocols in protocols.io to enhance the reproducibility of your results. Protocols.io assigns your protocol its own identifier (DOI) so that it can be cited independently in the future. For instructions see: https://journals.plos.org/plosone/s/submission-guidelines#loc-laboratory-protocols. Additionally, PLOS ONE offers an option for publishing peer-reviewed Lab Protocol articles, which describe protocols hosted on protocols.io. Read more information on sharing protocols at https://plos.org/protocols?utm_medium=editorial-email&utm_source=authorletters&utm_campaign=protocols.

As the corresponding author, your ORCID iD is verified in the submission system and will appear in the published article. PLOS supports the use of ORCID, and we encourage all coauthors to register for an ORCID iD and use it as well. Please encourage your coauthors to verify their ORCID iD within the submission system before final acceptance, as unverified ORCID iDs will not appear in the published article. *Only*  the individual author can complete the verification step; PLOS staff *cannot*  verify ORCID iDs on behalf of authors.

We look forward to receiving your revised manuscript.

Kind regards,

Grace Frempong Afrifa-Anane, Ph.D

Guest Editor

PLOS One

Journal Requirements:

If the reviewer comments include a recommendation to cite specific previously published works, please review and evaluate these publications to determine whether they are relevant and should be cited. There is no requirement to cite these works unless the editor has indicated otherwise.

Please review your reference list to ensure that it is complete and correct. If you have cited papers that have been retracted, please include the rationale for doing so in the manuscript text, or remove these references and replace them with relevant current references. Any changes to the reference list should be mentioned in the rebuttal letter that accompanies your revised manuscript. If you need to cite a retracted article, indicate the article’s retracted status in the References list and also include a citation and full reference for the retraction notice.

Additional Editor Comments:

1. Regarding the evidence of your variance inflation factor (VIF), I suggest you provide a Table that shows the results of the VIFs for the various variables, as well as the mean values. Provide this as a supplementary file.

2. Authors are also advised to use STROBE guidelines for reporting of observational studies.

[Note: HTML markup is below. Please do not edit.]

To ensure your figures meet our technical requirements, please review our figure guidelines: https://journals.plos.org/plosone/s/figures

You may also use PLOS’s free figure tool, NAAS, to help you prepare publication quality figures: https://journals.plos.org/plosone/s/figures#loc-tools-for-figure-preparation.

NAAS will assess whether your figures meet our technical requirements by comparing each figure against our figure specifications.

# Author comment: Author response to Decision Letter 3

**DOI:** 10.1371/journal.pone.0327266.r006

30 Apr 2026

The response to reviewer document has been attached on the paper

Attachment Submitted filename: Response_to_Reviewers._auresp_3.docx

# Editor report: Decision Letter 3

**DOI:** 10.1371/journal.pone.0327266.r007

**Authors:** Grace Afrifa-Anane, Grace Afrifa-Anane, Grace Afrifa-Anane, Grace Afrifa-Anane

12 May 2026

PONE-D-25-31448R3

Prevalence and associated factors of non-communicable diseases among men in Kenya: an analysis of the 2022 Kenya Demographic and Health Survey

PLOS One

Dear Dr. Asiimwe,

Thank you for submitting your manuscript to PLOS ONE. After careful consideration, we feel that it has merit but does not fully meet PLOS ONE’s publication criteria as it currently stands. Therefore, we invite you to submit a revised version of the manuscript that addresses the points raised during the review process.

Thank you for your revised document. However, further issues need to be addressed. Please see below:

1. “ Pg 1-2, LN 3, 35” The study design is not indicated in the title nor the abstract

2. Indicate the total number of respondents in Table 1

3. The  frequencies for the following variables: exercise minutes per week, past smoking variable,  number of hours per week seated, health status, do not sum up to the total number of respondent (N=14453).

4. Please, the justification for dropping the smoking tobacco variable in the VIF is not valid. I presume the variable was dropped because of an issue of multicollinearity. Meanwhile it was included in both regression analysis. Using both past smoking and tobacco use is problematic. Is the past smoking variable a sub-set of tobacco use? It is likely past users are included in category of those who answered no to tobacco use.  Why is there a drastic reduction in the frequency (if it’s not typo? Include both variables in the VIF for verification. I suggest you either drop past smoking or recode the two variables (past smoking and tobacco use) into one variable (never smoked, former smoker, current smoker).

5. Rename the supplementary files as S1 and S2

6. Recheck the line numbers reported in the Strobe checklist for accuracy.

Please submit your revised manuscript by Jun 26 2026 11:59PM If you will need more time than this to complete your revisions, please reply to this message or contact the journal office at plosone@plos.org. When you're ready to submit your revision, log on to https://www.editorialmanager.com/pone/ and select the 'Submissions Needing Revision' folder to locate your manuscript file.

Please include the following items when submitting your revised manuscript

- A letter that responds to each point raised by the academic editor and reviewer(s). You should upload this letter as a separate file labeled 'Response to Reviewers'.
- A marked-up copy of your manuscript that highlights changes made to the original version. You should upload this as a separate file labeled 'Revised Manuscript with Track Changes'.
- An unmarked version of your revised paper without tracked changes. You should upload this as a separate file labeled 'Manuscript'.

If you would like to make changes to your financial disclosure, please include your updated statement in your cover letter. Guidelines for resubmitting your figure files are available below the reviewer comments at the end of this letter.

If applicable, we recommend that you deposit your laboratory protocols in protocols.io to enhance the reproducibility of your results. Protocols.io assigns your protocol its own identifier (DOI) so that it can be cited independently in the future. For instructions see: https://journals.plos.org/plosone/s/submission-guidelines#loc-laboratory-protocols. Additionally, PLOS ONE offers an option for publishing peer-reviewed Lab Protocol articles, which describe protocols hosted on protocols.io. Read more information on sharing protocols at https://plos.org/protocols?utm_medium=editorial-email&utm_source=authorletters&utm_campaign=protocols.

As the corresponding author, your ORCID iD is verified in the submission system and will appear in the published article. PLOS supports the use of ORCID, and we encourage all coauthors to register for an ORCID iD and use it as well. Please encourage your coauthors to verify their ORCID iD within the submission system before final acceptance, as unverified ORCID iDs will not appear in the published article. *Only*  the individual author can complete the verification step; PLOS staff *cannot*  verify ORCID iDs on behalf of authors.

We look forward to receiving your revised manuscript.

Kind regards,

Grace Frempong Afrifa-Anane, Ph.D

Guest Editor

PLOS One

Journal Requirements:

If the reviewer comments include a recommendation to cite specific previously published works, please review and evaluate these publications to determine whether they are relevant and should be cited. There is no requirement to cite these works unless the editor has indicated otherwise.

Please review your reference list to ensure that it is complete and correct. If you have cited papers that have been retracted, please include the rationale for doing so in the manuscript text, or remove these references and replace them with relevant current references. Any changes to the reference list should be mentioned in the rebuttal letter that accompanies your revised manuscript. If you need to cite a retracted article, indicate the article’s retracted status in the References list and also include a citation and full reference for the retraction notice.

[Note: HTML markup is below. Please do not edit.]

[NOTE: If reviewer comments were submitted as an attachment file, they will be attached to this email and accessible via the submission site. Please log into your account, locate the manuscript record, and check for the action link "View Attachments". If this link does not appear, there are no attachment files.]

To ensure your figures meet our technical requirements, please review our figure guidelines: https://journals.plos.org/plosone/s/figures

You may also use PLOS’s free figure tool, NAAS, to help you prepare publication quality figures: https://journals.plos.org/plosone/s/figures#loc-tools-for-figure-preparation.

NAAS will assess whether your figures meet our technical requirements by comparing each figure against our figure specifications.

# Author comment: Author response to Decision Letter 4

**DOI:** 10.1371/journal.pone.0327266.r008

16 May 2026

We have attached a response to reviewers letter

Attachment Submitted filename: Response_to_Reviewers._auresp_4.docx

# Editor report: Decision Letter 4

**DOI:** 10.1371/journal.pone.0327266.r009

**Authors:** Grace Afrifa-Anane, Grace Afrifa-Anane, Grace Afrifa-Anane, Grace Afrifa-Anane, Grace Afrifa-Anane

24 May 2026

PONE-D-25-31448R4

Prevalence and associated factors of non-communicable diseases among men:  Across-sectional analysis of Kenya Demographic and Health Survey 2022 data.

PLOS One

Dear Dr. Asiimwe,

Thank you for submitting your manuscript to PLOS ONE. After careful consideration, we feel that it has merit but does not fully meet PLOS ONE’s publication criteria as it currently stands. Therefore, we invite you to submit a revised version of the manuscript that addresses the points raised during the review process.

Thank you for your response to the earlier comments. However, there are further statistical issues that need to be addressed.

1. How do you conclude or interpret your study if there were missing data and you included the variables without addressing those missing cases? How did you treat or address your missing cases? If you addressed them well, the sample size would have been equal , particularly at the bivariate level and regression levels. Please address the issue of missing cases in your data and redo the analysis. Please provide a flow chart of  how you arrived at your new sample size afterwards.
2. Also, Table two does not sum up to 14453 as indicated in the table (However, this sample will change after addressing the missing cases).  I expected to see a category of those who had no condition, or is your study amongst those who had a condition?
3. Importantly, respondents with two or more conditions sum up to 246 and not 275 as indicated (This is likely to change after addressing the missing cases.
4. The sample sizes are not equal in all stages of the analysis. Please indicate the sample size at each level of the analysis for readers to understand the variations in the methodology section.

Please submit your revised manuscript by Jul 08 2026 11:59PM. If you will need more time than this to complete your revisions, please reply to this message or contact the journal office at plosone@plos.org. When you're ready to submit your revision, log on to https://www.editorialmanager.com/pone/ and select the 'Submissions Needing Revision' folder to locate your manuscript file.

Please include the following items when submitting your revised manuscript:

- A letter that responds to each point raised by the academic editor and reviewer(s). You should upload this letter as a separate file labeled 'Response to Reviewers'.
- A marked-up copy of your manuscript that highlights changes made to the original version. You should upload this as a separate file labeled 'Revised Manuscript with Track Changes'.
- An unmarked version of your revised paper without tracked changes. You should upload this as a separate file labeled 'Manuscript'.

If you would like to make changes to your financial disclosure, please include your updated statement in your cover letter. Guidelines for resubmitting your figure files are available below the reviewer comments at the end of this letter.

If applicable, we recommend that you deposit your laboratory protocols in protocols.io to enhance the reproducibility of your results. Protocols.io assigns your protocol its own identifier (DOI) so that it can be cited independently in the future. For instructions see: https://journals.plos.org/plosone/s/submission-guidelines#loc-laboratory-protocols. Additionally, PLOS ONE offers an option for publishing peer-reviewed Lab Protocol articles, which describe protocols hosted on protocols.io. Read more information on sharing protocols at https://plos.org/protocols?utm_medium=editorial-email&utm_source=authorletters&utm_campaign=protocols.

As the corresponding author, your ORCID iD is verified in the submission system and will appear in the published article. PLOS supports the use of ORCID, and we encourage all coauthors to register for an ORCID iD and use it as well. Please encourage your coauthors to verify their ORCID iD within the submission system before final acceptance, as unverified ORCID iDs will not appear in the published article. *Only*  the individual author can complete the verification step; PLOS staff *cannot*  verify ORCID iDs on behalf of authors.

We look forward to receiving your revised manuscript.

Kind regards,

Grace Frempong Afrifa-Anane, Ph.D

Guest Editor

PLOS One

Journal Requirements:

1. If the reviewer comments include a recommendation to cite specific previously published works, please review and evaluate these publications to determine whether they are relevant and should be cited. There is no requirement to cite these works unless the editor has indicated otherwise.

2. Please review your reference list to ensure that it is complete and correct. If you have cited papers that have been retracted, please include the rationale for doing so in the manuscript text, or remove these references and replace them with relevant current references. Any changes to the reference list should be mentioned in the rebuttal letter that accompanies your revised manuscript. If you need to cite a retracted article, indicate the article’s retracted status in the References list and also include a citation and full reference for the retraction notice.

[Note: HTML markup is below. Please do not edit.]

[NOTE: If reviewer comments were submitted as an attachment file, they will be attached to this email and accessible via the submission site. Please log into your account, locate the manuscript record, and check for the action link "View Attachments". If this link does not appear, there are no attachment files.]

To ensure your figures meet our technical requirements, please review our figure guidelines: https://journals.plos.org/plosone/s/figures

You may also use PLOS’s free figure tool, NAAS, to help you prepare publication quality figures: https://journals.plos.org/plosone/s/figures#loc-tools-for-figure-preparation.

NAAS will assess whether your figures meet our technical requirements by comparing each figure against our figure specifications.

# Author comment: Author response to Decision Letter 5

**DOI:** 10.1371/journal.pone.0327266.r010

17 Jun 2026

We have attached a response to reviewer file

Attachment Submitted filename: Response_to_Reviewers._auresp_5.docx

# Editor report: Decision Letter 5

**DOI:** 10.1371/journal.pone.0327266.r011

**Authors:** Grace Afrifa-Anane, Grace Afrifa-Anane, Grace Afrifa-Anane, Grace Afrifa-Anane, Grace Afrifa-Anane, Grace Afrifa-Anane

1 Jul 2026

Prevalence and associated factors of non-communicable diseases among men:  Across-sectional analysis of Kenya Demographic and Health Survey 2022 data.

PONE-D-25-31448R5

Dear Dr.  Baptist Asiimwe,

We’re pleased to inform you that your manuscript has been judged scientifically suitable for publication and will be formally accepted for publication once it meets all outstanding technical requirements.

Within one week, you’ll receive an e-mail detailing the required amendments. When these have been addressed, you’ll receive a formal acceptance letter and your manuscript will be scheduled for publication.

An invoice will be generated when your article is formally accepted. Please note, if your institution has a publishing partnership with PLOS and your article meets the relevant criteria, all or part of your publication costs will be covered. Please make sure your user information is up-to-date by logging into Editorial Manager at Editorial Manager® and clicking the ‘Update My Information' link at the top of the page. For questions related to billing, please contact billing support.

If your institution or institutions have a press office, please notify them about your upcoming paper to help maximize its impact. If they’ll be preparing press materials, please inform our press team as soon as possible -- no later than 48 hours after receiving the formal acceptance. Your manuscript will remain under strict press embargo until 2 pm Eastern Time on the date of publication. For more information, please contact onepress@plos.org.

Kind regards,

Grace Frempong Afrifa-Anane, Ph.D

Guest Editor

PLOS One

Additional Editor Comments (optional):

Reviewers' comments:

# Editor report: Acceptance letter

**DOI:** 10.1371/journal.pone.0327266.r012

**Authors:** Grace Afrifa-Anane, Grace Afrifa-Anane, Grace Afrifa-Anane, Grace Afrifa-Anane, Grace Afrifa-Anane, Grace Afrifa-Anane

PONE-D-25-31448R5

PLOS One

Dear Dr. Asiimwe,

I'm pleased to inform you that your manuscript has been deemed suitable for publication in PLOS One. Congratulations! Your manuscript is now being handed over to our production team.

At this stage, our production department will prepare your paper for publication. This includes ensuring the following:

* All references, tables, and figures are properly cited

* All relevant supporting information is included in the manuscript submission,

* There are no issues that prevent the paper from being properly typeset

You will receive further instructions from the production team, including instructions on how to review your proof when it is ready. Please keep in mind that we are working through a large volume of accepted articles, so please give us a few days to review your paper and let you know the next and final steps.

Lastly, if your institution or institutions have a press office, please let them know about your upcoming paper now to help maximize its impact. If they'll be preparing press materials, please inform our press team within the next 48 hours. Your manuscript will remain under strict press embargo until 2 pm Eastern Time on the date of publication. For more information, please contact onepress@plos.org.

You will receive an invoice from PLOS for your publication fee after your manuscript has reached the completed accept phase. If you receive an email requesting payment before acceptance or for any other service, this may be a phishing scheme. Learn how to identify phishing emails and protect your accounts at https://explore.plos.org/phishing.

If we can help with anything else, please email us at customercare@plos.org.

Thank you for submitting your work to PLOS ONE and supporting open access.

Kind regards,

PLOS ONE Editorial Office Staff

on behalf of

Dr. Grace Frempong Afrifa-Anane

Guest Editor

PLOS One
